# Family of Quasi-Isotropic Mn^II^ and Mn_2_^II^ Complexes Exhibiting Slow Relaxation of the Magnetization

**DOI:** 10.1021/acs.inorgchem.4c02826

**Published:** 2024-10-16

**Authors:** Evangelos Pilichos, Mercè Font-Bardia, Gabriel Aullón, Júlia Mayans, Albert Escuer

**Affiliations:** † Departament de Química Inorgànica i Orgànica, Secció Inorgànica and Institute of Nanoscience and Nanotecnology, Universitat de Barcelona, Marti i Franques 1-11, Barcelona 08028, Spain; ‡ Departament de Mineralogia, Cristal·lografia i Dipòsits Minerals, Universitat de Barcelona, Martí Franqués s/n, 08028 Barcelona, Spain; § Unitat de Difracció de R-X. Centre Científic i Tecnològic de la Universitat de Barcelona, Solé i Sabarís 1-3., 08028 Barcelona, Spain; ∥ Departament de Química Inorgànica i Orgànica, Secció Inorgànica and Institut de Química Teòrica i Computacional, Universitat de Barcelona, 08028 Barcelona, Spain

## Abstract

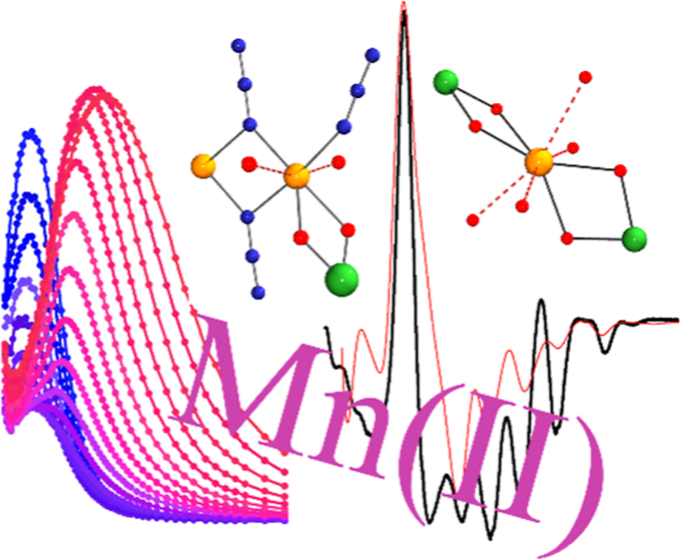

Slow relaxation of magnetization has been studied for a family of mononuclear Mn^II^ complexes and one ferromagnetic dinuclear system, all of them presenting very weak anisotropy. Complexes with formula [{NiL1Mn(H_2_O)_2_(MeOH)}{NiL1}_2_](ClO_4_)_2_ (**1**), [Mn{NiL1}_2_](ClO_4_)_2_ (**2**), [Mn{NiL2}_2_](ClO_4_)_2_ (RR–L2^2–^, **3**RR, SS–L2^2–^, **3**SS), [Mn{NiL3}_2_](ClO_4_)_2_ (RR–L3^2–^, **4**RR, SS–L3^2–^, **4**SS) and (μ_1,1_-N_3_)_2_[Ni_2_Mn_2_(L1)_2_(N_3_)_2_] (**5**) are derived from compartmental Schiff bases, in which the Ni^II^ environment is square planar and thus diamagnetic. All of the systems have been structurally and magnetically characterized. Zero field splitting (*D*) values for the Mn^II^ cations have been obtained from EPR spectroscopy and NEVPT2 calculations. The slow relaxation of the magnetization for **1**–**5** has been studied by means of ac magnetometry and rationalized on the basis of their low, but not zero, anisotropy, providing the first example of a polynuclear Mn^II^ complex, with *S* = 5 ground state, exhibiting slow relaxation.

## Abstract 2

Slow relaxation of the magnetization has been found for a family of quasi-isotropic Mn^II^ complexes, which includes the first case for a homometallic polynuclear Mn^II^ system.

## Introduction

The occurrence of magnetic anisotropy plays a major role in the magnetic properties exhibited by low-dimensional systems, known as single molecule/ion magnets (SMM/SIM).^1,2^ Magnetic anisotropy is the dependence of magnetic properties on the spatial directions under the application of a magnetic field, and its control is still a challenging issue for synthetic chemists because it is highly dependent on the geometry around the spin carriers.^3^ This direct relation between the coordination environment around the cation with the occurrence of magnetic anisotropy makes syntheses extremely important when trying to enhance the magnetic properties of the working systems.^4^ Even with the possibility of directed syntheses and rational design in selecting the adequate ligands and cations, serendipity is nowadays very important in the production of low dimensional coordination compounds for molecular applications. The correct alignment of the magnetic anisotropy improves the slow magnetic relaxation of the material to use it in different fields like spintronics,^5,6^ magnetic memory storage, or quantum computation.^7^ The origin of magnetic anisotropy in d cations is related to their electronic structure, which derives from the crystal field terms, which are their main source of magnetic anisotropy. This phenomenon has been widely employed to generate, for example, a large number Mn^III^ or Co^II^ SMM/SIMs.^8,9^

In contrast with the usual SMM/SIMs, for which a large anisotropy becomes a requirement, slow relaxation of the magnetization (SRM) for the cations with half-filled shell (either d^5^ transition or f^7^ lanthanoid cations), a priori, is not expected due to its negligible zero field splitting and the lack of double-well and the derived barrier for the SRM. However, this paradigm has been broken because recently, a scarce number of magnetically isotropic slow-relaxing compounds derived of d^5^ or f^7^ half-filled configurations have appeared in the literature, mainly derived from trivalent gadolinium^10−17^ and more rarely from divalent manganese.^18−25^ The possibility of slow relaxation in isotropic systems is fascinating and remains poorly studied despite some hypotheses about the origin of the relaxation, which could be related with very specific magnitudes of the value of the axial zero field splitting (*D*).^14,16^ A different case is the high spin Fe^III^ cation because, in spite of its ground ^6^*A*_1g_ term, it exhibits well-defined barriers for the reversal of the magnetization in mononuclear^3^ or polynuclear^26^ systems due to the presence of low-lying quartet or doublet states.^27^ This emerging property derived from the zero orbital momentum (*L* = 0) has been recently explored for Gd^III^ in combination with pulsed-EPR experiments and provides an opportunity as qubits due to its long spin relaxation time.^28^

The d^5^ high spin Mn^II^ cation (^6^*A*_1g_ term in high symmetry environments such *O*_h_) with orbital contribution *L* = 0 becomes isotropic, and for an ideal topology, its *D* value is zero, and thus, SRM should be discarded according the usual rules for SMM/SIM, in which the relaxation barrier is related with the magnitude of the axial zero field splitting and the square of the total spin state of the system (DS^2^ – 1/4 = 6*D* in this case). However, the *m*_s_ degeneracy can be broken, reducing the symmetry by employing the adequate ligands in *O*_h_ coordination and/or changing the coordination number. These factors have been studied using high field EPR by Duboc et al. for a series of hexa- and penta-coordinated Mn^II^ complexes establishing the dependence of the *D* value with the donor atoms (|*D*_N,O_| < |*D*_Cl_| ≪ |*D*_Br_| < |*D*_I_|) with absolute values ranging between less than ∼0.2 cm^–1^ for N, O donors up to ∼1 cm^–1^ for heavy donors like iodide.^29,30^ In all cases, larger *D* values were found for pentacoordination in comparison with the octahedral environment. The plastic coordination sphere of the Mn^II^, derived from the zero ligand field contribution of the high spin d^5^ configuration, makes it easy to reach coordination numbers between 4 and 8 by the adequate selection of the organic ligands and can be made to induce some degree of anisotropy.

**Chart 1:**
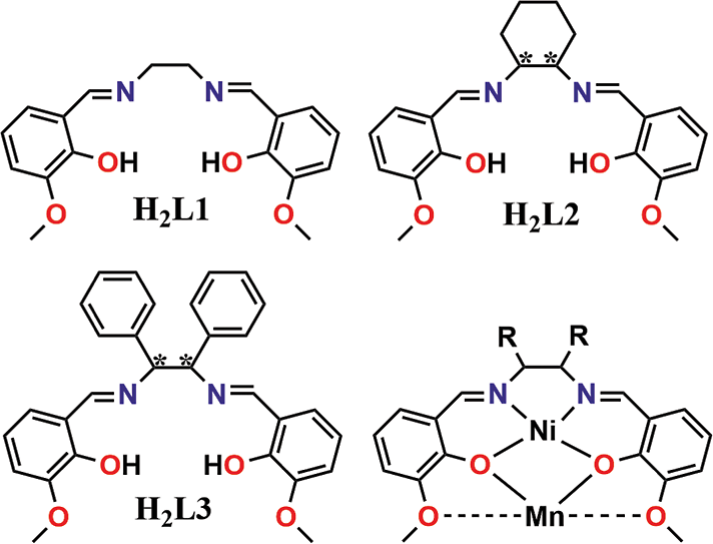
Schematic plot of the ligands employed in this work showing the coordination found in compounds **1–5** that links the Ni^II^ cation in the inner N_2_O_2_ cavity and the Mn^II^ cation in the external cavity of the Schiff basea

With the aim to design a series of Mn^II^ complexes with a strong distortion of their coordination environment, able to induce a moderate anisotropy, we selected the hexadentate Schiff bases depicted in Chart 1, which provide three different kinds of donors, which can interact in a different manner with the cations. To act as a spacer, leading to a better isolation of the paramagnetic Mn^II^ cations, the inner N_2_O_2_ cavity was occupied with one Ni^II^ cation in a square planar environment that becomes diamagnetic. Cascade reaction of the corresponding Schiff base with Ni^II^, followed by manganese perchlorate, allowed for the isolation of the tri- and tetranuclear complexes [{NiL1Mn(H_2_O)_2_(MeOH)}{NiL1}_2_](ClO_4_)_2_ (**1**), [Mn{NiL1}_2_](ClO_4_)_2_ (**2**), [Mn{NiL2}_2_](ClO_4_)_2_ (RR–L2^2–^, **3**RR, SS–L2^2–^, **3**SS) and [Mn{NiL3}_2_](ClO_4_)_2_ (RR–L3^2–^, **4**RR, SS–L3^2–^, **4**SS), which contains a unique paramagnetic cation. These systems exhibit SRM in all cases, and their magnetic properties have been studied and related with their degree of anisotropy.

a Dotted bonds indicate weak interaction. Asterisks denotes chiral centers.

The SRM for isotropic systems has been studied on mononuclear systems with the exception of some Cu^II^–Gd^III^ dinuclear complexes^31,32^ and several Cu^II^/Mn^II^ systems with different nuclearities and spin ground state, recently reported by us,^33,34^ and for one trimeric homometallic Mn^II^ complex with the unusual heterospin 1/2–5/2–1/2 distribution.^35^ In light of the new and interesting properties of the complexes **1–4**, we prepared the relevant, previously synthesized complex, (μ_1,1_-N_3_)_2_[Ni_2_Mn_2_(L1)_2_(N_3_)_2_] (**5**), which contains a double end-on azido bridge and ferromagnetic response,^36^ which was revealed to be the first high spin polynuclear Mn^II^ complex exhibiting SRM and has become the larger spin reported to date for slow relaxing isotropic systems.

## Experimental Section

### Physical Measurements

The yield of the **1–5** complexes is referred to as the well-formed crystalline fraction that has been employed in the instrumental measurements. Magnetic susceptibility measurements were carried out on pressed polycrystalline samples with a MPMS7 Quantum Design susceptometer working in the range 30–300 K under magnetic fields of 0.3 T and under a field of 0.03 T in the 30–2 K range to avoid saturation effects at low temperature. Diamagnetic corrections were estimated from Pascal Tables.^37^ Infrared spectra (4000–400 cm^–1^) were recorded from KBr pellets on a Bruker IFS-125 FT-IR spectrophotometer. ECD spectra were recorded in methanolic solutions with a Jasco-815 spectropolarimeter. EPR spectra of polycrystalline samples were recorded at 4 K with a Bruker ELEXYS E580 spectrometer equipped with a helium continuous-flow cryostat. Powder X-ray diffraction was performed with a PANalytical X’Pert PRO MPD θ/θ powder diffractometer of 240 mm of radius, in a configuration of convergent beam with a focalizing mirror and a transmission geometry with flat samples sandwiched between low absorbing films and Cu Kα radiation (λ = 1.5418 Å).

### X-ray Crystallography

Red needles (**NiL3**, **1,** and **3**SS) and red prism-like specimens (**2**, **4**RR, and **4**SS) were used for the single crystal X-ray crystallographic analysis. The X-ray intensity data were measured on a D8 Venture system equipped with a multilayer monochromator and a Mo microfocus. The frames were integrated with the Bruker SAINT software package using a narrow-frame algorithm. The structures were solved and refined using the Bruker SHELXTL Software Package.^38^ Crystal data and refinement details for complexes **1**, **2**, **3**SS, **4**RR, and **4**SS are summarized in Table S1. Complex **5** was characterized by powder X-ray diffraction by comparison with the reported structure of CCCD-CIBPAF, as shown in Figure S1. The structure of the monomeric precursor **NiL3**, less relevant for the target of this work, is only described in the Supporting Information, Tables S2 and S3 and Figure S2. Further crystallographic details can be found in the corresponding CIF files provided in the Supporting Information.

### Theoretical Calculations

The *n*-electron valence perturbation theory (CASSCF-NEVPT2)^39−41^ calculations were performed with the ORCA code (version 5.0.3)^42^ using experimental geometries. Scalar relativistic effects were included using the second order Douglas–Kroll–Hess (DKH) method.^43^ Polarized basis sets having triple-ξ quality developed by Ahlrichs and co-workers were used for all elements (def2-TZVPP).^44−46^ The active space contains five electrons on five active 3d molecular orbitals, and the ZFS tensor *D* of zero-field splitting Hamiltonian was extracted including spin–orbit coupling correction.^47,48^

### Synthesis of the [NiL(H_2_O)] Precursors, L = L1^2–^, L2^2–^, and L3^2–^

The precursors were synthesized following a modification of the reported procedure.^49^*o*-vanillin (0.8 mmol/0.121 g) was added to a mixture of nickel(II) acetate tetrahydrate (0.4 mmol/0.100 g) and 0.4 mmol of the corresponding diamine (ethylenediamine 0.024 g, (*R*,*R*- or *S*,*S*)-1,2-diaminocyclohaxane) 0.045 g, and (*R*,*R* or *S*,*S*)-1,2-diphenylethylenediamine 0.085 g in 15 mL of H_2_O/MeOH (1:2). The reaction solutions were heated for 30 min at 80 °C in an Anton Paar Monowave-300 microwaves furnace. On cooling, a reddish precipitate of the corresponding complex [Ni^II^L(H_2_O)] was formed with a ∼90% yield, collected, and dried under vacuum. Crystals of [Ni^II^L3(H_2_O)] (**NiL3**) were isolated from the corresponding filtered solution by slow evaporation.

### [{NiL1Mn(H_2_O)_2_(MeOH)}{NiL1}_2_](ClO_4_)_2_·MeOH (1·MeOH) and [Mn{NiL1}_2_](ClO_4_)_2_·2.5CH_2_Cl_2_·0.5MeOH (2·2.5CH_2_Cl_2_·0.5MeOH)

Caution! Perchlorates are potentially explosive, and the syntheses should be performed in low amounts and handled with caution.

A suspension of [Ni^II^L1(H_2_O)] (0.25 mmol, 0.100 g) in 15 mL of dichloromethane/methanol (3:1) was added to 5 mL of a methanolic solution of manganese perchlorate hydrate (0.25 mmol/0.091 g). The reaction mixture was left in a microwave furnace for 15 min at 70 °C. The obtained orange solution was layered with diethyl ether and left for crystallization. In 1 week, well-shaped red crystals, suitable for X-ray diffraction, were obtained. The crystallization gives two kind of crystals, needles that correspond to complex **1** and prismatic crystals that correspond to complex **2** in similar amounts that were separated manually. Yield: ∼20% for both compounds. Anal. Calcd for C_56_H_66_Cl_2_MnN_6_Ni_3_O_24_ (**1**·MeOH) C, 44.57; H, 4.41; N, 5.57%. Found: C, 44.1; H, 4.46; N, 5.42%. Anal. Calcd for C_36.5_H_38_Cl_2_MnN_4_Ni_2_O_16.5_ (**2**·0.5MeOH, dried) C, 42.16; H, 3.68; N, 5.39%. Found: C, 42.6; H, 3.7; N, 5.6%. IR spectra are reported in Figure S3.

### [Mn(NiL2)_2_](ClO_4_)_2_ (**3**SS and **3**RR) and [Mn(NiL3)_2_](ClO_4_)_2_ (**4**RR and **4**SS)

The two complexes were synthesized following the same procedure that was used for **1** and **2** but employing 0.25 mmol of the corresponding amount of [Ni^II^L(H_2_O)] (L = L2^2–^, 0.115 g; L3^2–^, 0.140 g). Crystals suitable for X-ray determination for **3** were obtained by layering with diethyl ether. Employing ligands H_2_L2 and H_2_L3, only one kind of red crystal was obtained. Yield: similar for both complexes ∼60%. Anal. Calcd for C_60_H_52_Cl_2_MnN_4_Ni_2_O_16_ (**3**, dried) C, 54.25; H, 3.94; N, 4.22%. Found for **3**RR: C, 54.0; H, 4.0; N, 4.3%. Found for **3**SS: C, 54.0; H, 3.8; N, 4.2%. Anal. Calcd for C_45_H_52_Cl_2_MnN_4_Ni_2_O_17_ (4·MeOH, dried) C, 46.43; H, 4.50; N, 4.81%. Found for **4**RR: C, 46.8; H, 4.4; N, 4.6%. Found for **4**SS: C, 46.2; H, 4.3; N, 4.6%. IR spectra are reported in Figure S3. Electronic circular dichroism (ECD) spectra, similar to previously related systems containing the same ligands^34,50^ are reported in Figure S4.

### (μ_1,1_-N_3_)_2_[Ni_2_Mn_2_(L1)_2_(N_3_)_2_] (**5**)

Caution! Azides are potentially explosive, and the syntheses should be performed in small amounts and handled with caution. The complex was synthesized by a modified method of the reported synthesis.^36^ The complex was prepared following the same procedure as complexes **1** and **2,** but adding NaN_3_ (0.4 mmol/0.026 g) to the [Ni(L1)(H_2_O)]/Mn(ClO_4_)_2_·6H_2_O dissolution. After 2 days, well-shaped orange-red crystals suitable for X-ray diffraction were formed by layering with diethyl ether. Yield: 40%. Anal. Calcd for C_36_H_36_Mn_2_N_16_Ni_2_O_8_; C, 41.26; H, 3.46; N, 21.38%. Found: C, 41.0; H, 3.6; N, 21.7%. IR spectra are reported in Figure S3.

## Results and Discussion

### Structural Description

#### [{NiL1Mn(H_2_O)_2_(MeOH)}{NiL1}_2_](ClO_4_)_2_·MeOH (1·MeOH)

**Figure 1:**
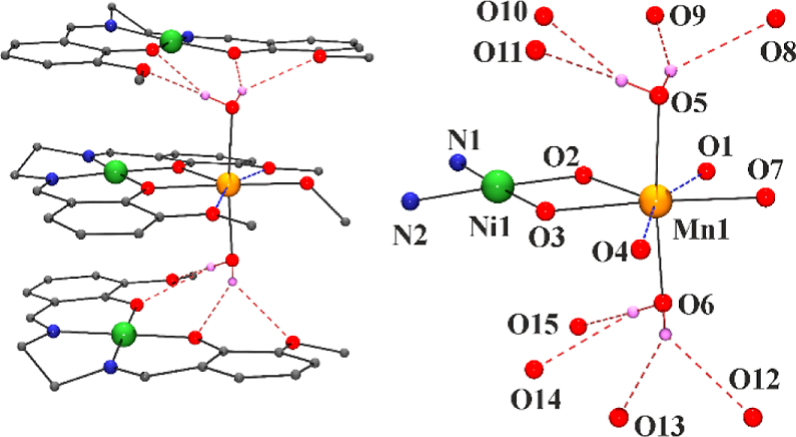
(Left) View of the molecular unit of **1**. (Right) Labeled core of the central dimeric unit. Blue dashed bonds with O_1_ and O_4_ denotes the large bond distances. Red dashed bonds show the H-bonds that link the dimeric [NiL1Mn(H_2_O)_2_(MeOH)]^2+^ fragment with the two neutral [NiL1] units.

Compound **1** consists of one [NiL1Mn(H_2_O)_2_(MeOH)]^2+^ dinuclear complex linked by means of H-bonds to two neutral [NiL1] units and two perchlorate counteranions, as shown in Figure 1. Main bond parameters are summarized in Table S4.

The heterodinuclear unit consists of one Ni^II^ cation placed in the N_2_O_2_ cavity of the Schiff base and one Mn^II^ cation linked by the *O*-diphenoxo donors. The Ni^II^ cations show a square planar environment with Ni–N and Ni–O bond distances of ∼1.85 Å, corresponding to this coordination. The coordination of the manganese cations consists of two bridging *O*-phenoxo donors, one methanol and two water molecules placed in trans arrangement. In addition, the two *O*-methoxo donors O_1_ and O_4_ weakly interact with the Mn^II^ cation, with Mn–O distances larger than 2.5 Å. Thus, the environment around the Mn^II^ cation can be envisaged as a MnO_5_ or MnO_7_ if the large Mn–O_1_ and Mn–O_4_ contacts are taken into account. SHAPE analysis^51^ of the coordination environment of the manganese cation shows large deviation from the trigonal bipyramid if only the short bonds are taken into account and a CShM of 1.58 for the pentagonal bipyramidal polyhedron if the large bonds with the methoxo donors are included, as shown in Figure S5.

The coordinated water molecules link the neutral [NiL1] fragments by means of bifurcated H-bonds with the four O atoms of the Schiff bases, as shown in Table S5, being shorter than those that involve the *O*-phenoxo donors, resulting in a sandwich structure. The clusters of **1** are pillared, forming chains of sandwiches that isolate the paramagnetic Mn^II^ cations with Mn···Mn distances along the chain of 11.890(1) Å and interchain distances of 11.461(1) Å, Figure S6.

#### [Mn{NiL1}_2_](ClO_4_)_2_·2.5CH_2_Cl_2_·0.5MeOH (2·2.5CH_2_Cl_2_·0.5MeOH)

**Figure 2:**
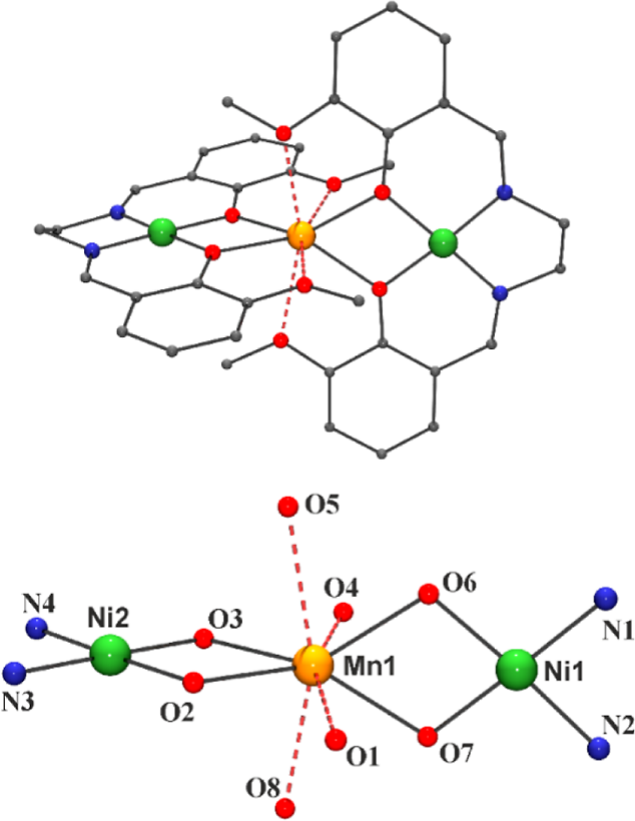
Top) View of the molecular structure of the trinuclear complex **2**. (Bottom) A labeled view of the Mn^II^ environment. Red dashed Mn–O bonds are those involving large distances.

The structure of the trinuclear complex **2** consists of two [NiL1] neutral fragments that coordinate one Mn^II^ cation by means of the *O*-phenoxo donors that act as a bridge between the Ni^II^ and the Mn^II^ cations, as shown in Figure 2. Main bond parameters are given in Table S6.

The Ni^II^ cations exhibit a square planar environment, with bond parameters similar to the case of complex **1**. The coordination sphere of the manganese cation is unusual, showing four short Mn–O_phenoxo_ bond distances (∼2.17 Å) and four larger Mn–O_methoxo_ bond distances larger than 2.4 Å. The MnO_4_ (*O*-_phenoxo_) environment can be described as a very distorted tetrahedron elongated along the Ni···Mn···Ni axis. The O_2_–Mn–O_3_ and O_6_–Mn–O_7_ bond angles (67.4 and 67.7°) are much lower than 109.5° and the four remainder O–Mn–O bond angles are consequently enlarged, taking values ranging between 129.00(3) and 136.88(3)°. The main planes of the two Schiff bases are roughly perpendicular with a dihedral angle between the mean Ni_1_–O_6_–O_7_–Mn and Ni_2_–O_2_–O_3_–Mn planes of 84.8°.

In addition, the environment of the Mn^II^ cation includes four large contacts forming a “belt” of the O-donors perpendicular to the main molecular axis. These four atoms are not in the same plane, showing a weak tetrahedral distortion. SHAPE analysis of the coordination polyhedron around the manganese cation shows very large deviations to the closest polyhedra due to the low bite of the Schiff base and the irregular distribution of bond lengths (CShM of 3.03 for the triangular dodecahedron or 3.32 for the biaugmented trigonal prism) and thus, its symmetry should be assumed as *C*_1,_ as shown in Figure S5. Weak intermolecular π–π stacking involving the pyridinic rings determines a zigzag arrangement of trimers in the network with a minimum intermolecular Mn···Mn distance of 8.993(1) Å, as shown in Figure S7-top.

#### [Mn(NiL2)_2_](ClO_4_)_2_·2CH_2_Cl_2_·MeOH (3SS·2CH_2_Cl_2_·MeOH)

**Figure 3:**
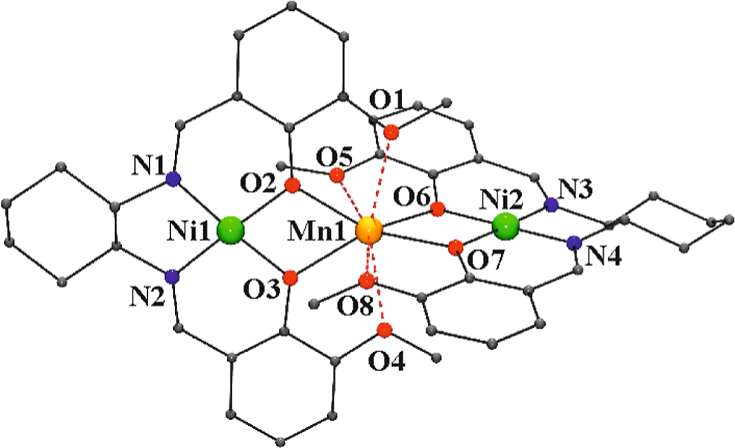
Partially labeled plot of the molecular structure of complex **3**. Red dashed Mn–O bonds are those involving large bond distances.

The structure of complex **3**SS is similar in their general trends to that of the previously described complex **2**. A plot of the molecular structure is shown in Figure 3, and the main bond parameters are summarized in Table S7. The coordination environment and bond parameters for the cations are close to those of the previous compound. The CShM value of 2.72 with respect to the closest polyhedron (triangular dodecahedron) evidences a strongly distorted environment, as shown in Figure S5. The main planes of the two Schiff basis are also roughly perpendicular with a dihedral angle between the mean Ni_1_–O_2_–O_3_–Mn and Ni_2_–O_6_–O_7_–Mn planes of 84.02°, and the main difference is related with the packing of the trimers, which in this case form linear chains mediated by π–π stacking of the pyridinic rings, resulting in a slightly shorter Mn···Mn intermolecular distance of 8.444(1) Å, as shown in Figure S7-middle.

#### [Mn(NiL3)_2_](ClO_4_)_2_ (**4**RR and **4**SS)

**Figure 4:**
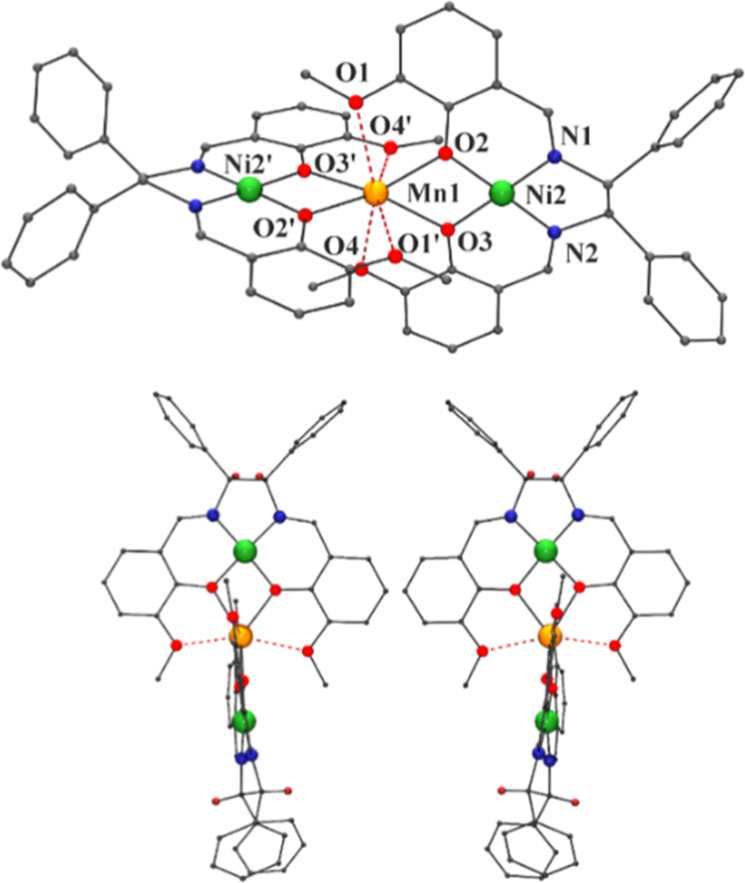
Top, partially labeled plot of the molecular structure of complex **4**RR. Bottom, a view of the specular molecules **4**RR and **4**SS. Red dashed Mn–O bonds are those involving large bond distances.

The structures of complexes **4** are similar in their general trends to those of the previously described compounds **2** and **3**. A plot of the molecular structure of **4**SS is shown in Figure 4-top, and the main bond parameters are summarized in Table S8. The coordination environment and bond parameters for the cations are close to those of the previous compounds, but the molecule is more symmetrical due to a *C*_2_ axis that passes through the manganese atom. The CShM value of 2.85 with respect to the closest polyhedron (triangular dodecahedron) evidences a strongly distorted environment, as shown in Figure S5. The dihedral angles between the mean Ni–O_2_–O_3_–Mn and Ni′-O_2_′-O_3_′-Mn planes for the two crystallographically nonequivalent trimers present in the unit cell are 81.7(2) and 85.6(1)°. The trimers form linear zigzag chains mediated by π–π stacking of the phenyl rings, resulting in an intrachain Mn···Mn intermolecular distance of 14.438(1) Å and a shorter interchain distance of 9.143(1) Å, as shown in Figure S7-bottom. Resolution of the crystal structure of the **4**RR complex shows the expected mirror image structure, as shown in Figure 4-bottom.

#### (μ_1,1_-N_3_)_2_[Ni_2_Mn_2_(L1)_2_(N_3_)_2_] (**5**)

**Figure 5:**
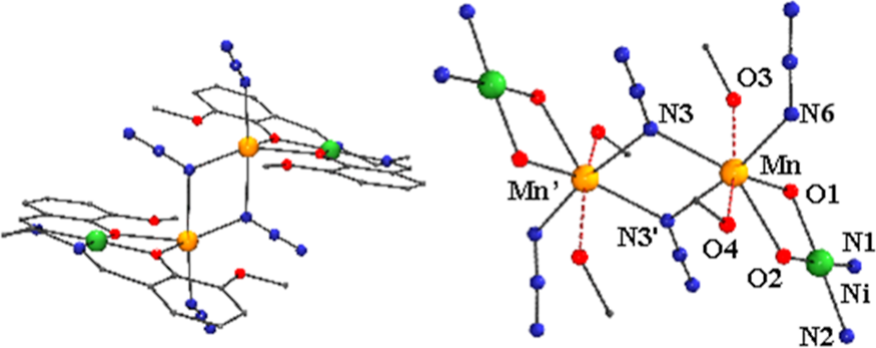
(Left) View of the molecular unit and (right) partially labeled core of complex **5**, plotted from CCCD-CIBPAF data. Red dashed Mn–O bonds are those involving large bond distances.

The structure of the centrosymmetric dimeric complex **5** was previously reported, and a complete description can be found in the original paper.^36^ In short, the structure consists of two [NiMnL1(N_3_)]^+^ fragments with the same arrangement of Ni^II^ in the inner and Mn^II^ in the outer ligand cavities, described in the previously described complexes, which are linked by a double end-on azido bridge between the Mn^II^ cations, as shown in Figure 5. Relevant bond parameters in relationship to the magnetic properties are summarized in Table S9. The Ni^II^ cations are placed in the inner cavity of the ligands, showing a square planar environment, whereas the Mn^II^ cations link the four O-donors from the Schiff bases, one coplanar azido, and two axial azido ligands, resulting in a distorted pentagonal bipyramid coordination. The Mn–N–Mn bond angle of 103.78(7)° lies in the usual range of values for double end-on azido bridges.^52^ In a way similar to complex **1**, the closest polyhedron to the environment around the Mn^II^ cation is a strongly distorted pentagonal bipyramid with a CShM value off 2.67, as shown in Figure S5.

### Comment to the Syntheses

The syntheses of the reported complexes are sensitive to the solvents and substituents on Schiff bases. Complex **1** is similar to a previously reported complex with *N*,*N*′-ethylenebis(3-ethoxysalicylaldimine instead the methoxy H_2_L1 ligand.^37^ In that case, cocrystallization of trinuclear {NiMnNi} species was not reported, and the complex coordinates a water ligand instead a methanol molecule. The changes of solubility due to the hexane or diphenyl substituents in the Schiff bases H_2_L2 and H_2_L3 are the most reasonable reason that uniquely allows trinuclear complexes. Trials to obtain new dinuclear systems similar to **5** with H_2_L2 and H_2_L3 instead H_2_L1 including azido ligands were unsuccessful, and poorly crystalline products were obtained in the two cases. A partial resolution of the structure of the compound obtained from H_2_L2 evidenced a polymeric arrangement of bridging azido ligands instead of the dimeric units, but a full characterization was not possible.

### Static Magnetic Properties and EPR Measurements for Complexes **1–4**

**Figure 6:**
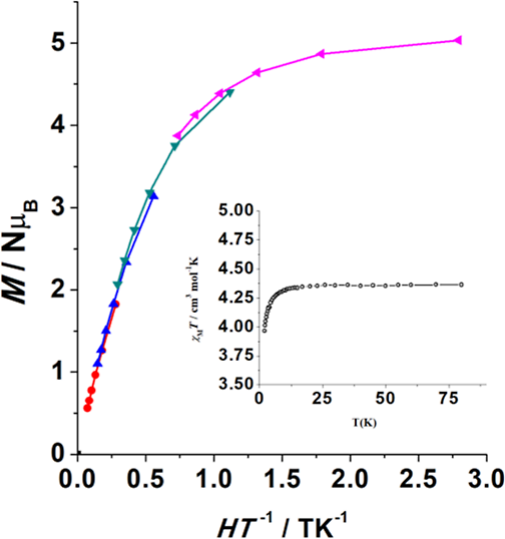
Reduced magnetization plot in the 1.8–6.8 K with 1 K increment for complex **1**. Inset χ_M_*T* plot showing the Curie response down 10 K. The similar plots for complexes **2**, **3**SS, and **4**SS are shown in Figure S8.

The experimental magnetic data and EPR spectra were analyzed with PHI^53^ and Easy Spin^54^ software, respectively. The dc magnetic susceptibility of **1–4** was measured on polycrystalline samples in the temperature range of 2–100 K. The four complexes show a Curie-law response with constant χ_M_*T* values close to the expected value for an isotropic *S* = 5/2 spin of 4.375 cm^3^·mol^–1^·K, as shown in Figures 6 and S8-insets. Compounds **3**SS and **4**SS follow a Curie law in all the temperature ranges, whereas for **1** and **2,** the χ_M_*T* value shows a slight decrease at very low temperature (below 10 K), reaching a value of 3.96 cm^3^·mol^–1^·K for **1** and 3.82 cm^3^·mol^–1^·K for **2**. Magnetization experiments show a saturation value close to the expected value of 5.0 Nμ_β_. Reduced magnetization measurements between 1.8 and 6.8 K show quasi superimposable plots, indicating a very weak anisotropy for **1** and **2** and superimposable plots for **3**SS and **4**SS, as shown in Figures 6 and S8.

The decay of the χ_M_*T* plots for **1** and **2** could be due to zero field splitting (ZFS) or intermolecular interactions, but these parameters are correlated and they should be assigned on the basis of combined measurements. Fit of the χ_M_*T* data can be equally simulated with *D* values of ∼1 cm^–1^ or intermolecular interactions z*J*′ with values of ∼0.015 cm^–1^. On the one hand, *D* values close to 1 cm^–1^ are not realistic for the Mn^II^ cation in the O-donor environment, and on the other hand, the quasi negligible anisotropy indicated by the reduced magnetization measurements is not compatible with such *D* values, and thus, the χ_M_*T* decay should be mainly assigned to very weak intermolecular interactions. The Curie behavior and reduced magnetization response for **1–4** confirms very low anisotropy for the Mn^II^ cations that cannot be calculated properly from magnetometry, as shown in Figures 6 and S8.

**Figure 7:**
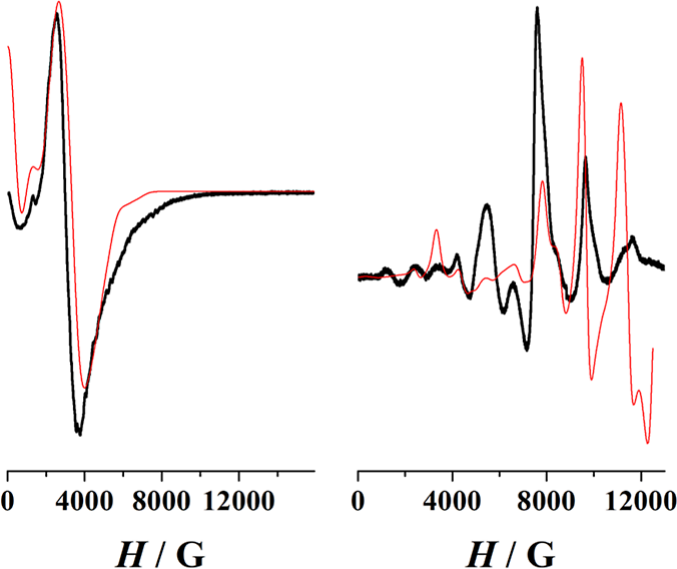
(Left) X-band EPR spectrum for complex **1**. (Right) Q-band EPR spectrum for complex **4**SS. Red lines show the best fits of the experimental data.

In order to obtain reliable information on the anisotropy parameters, the EPR spectra were recorded for complex **1** (X-band) and, taking into account the quasi identical environment for **2–4**, for the representative complex **4** (Q-band). Complex **1** yielded a broad spectrum that was equally simulated with a *D* = 0.080 cm^–1^ and an *E*/*D* ratio of 0.20 or *D* = −0.084 cm^–1^ and an *E*/*D* ratio of 0.20, which are in agreement with the structural and static magnetic measurements data, confirming its weak *D* value and indicating moderate rhombicity, as shown in Figure 7-left.

Complex **4** shows a complex spectrum in a wide range of field, as shown in Figure 7-right, which could be equally fitted with a *D* value of 0.205 cm^–1^ and a low *E*/*D* ratio of 0.036 or negative values of *D* = −0.207 cm^–1^ and *E*/*D* = 0.035, in agreement with the axially elongated tetrahedron close to the ideal *D*_2d_ symmetry, as shown in Figure 2. In light of the uncertainty on the sign of *D* from the EPR spectra, theoretical calculations were performed (see the Computational Data section).

### Dynamic Magnetic Properties

#### Complexes **1–4**

The ac response for mononuclear complexes of isotropic cations has been reported for the Gd^III^ cation^10−17^ (*f*^7^, *L* = 0) and some isolated Mn^II^ complexes.^18−24^ Its out-of-phase behavior is characterized by the presence of two kind of signals, one of them at low temperature for the lower frequencies (LFT), which usually is field-dependent but frequency- and temperature-independent. According to the τ = (2πν)^−1^ expression, the LFT process provides large relaxation times (τ > 10^–2^ s). The second block of signals appear at a higher temperature: they are always frequency- and temperature-dependent (HFT) and promote relaxation times in the usual 10^–5^–10^–8^ s range. The overlap of the two processes determines the treatment of the experimental data: the relaxation times for the LFT process can usually be directly evaluated from the maxima in the χ″_M_(ν) plot, whereas for the HFT process, it is recommended to fit the Argand plot discarding the lower temperatures.

**Figure 8:**
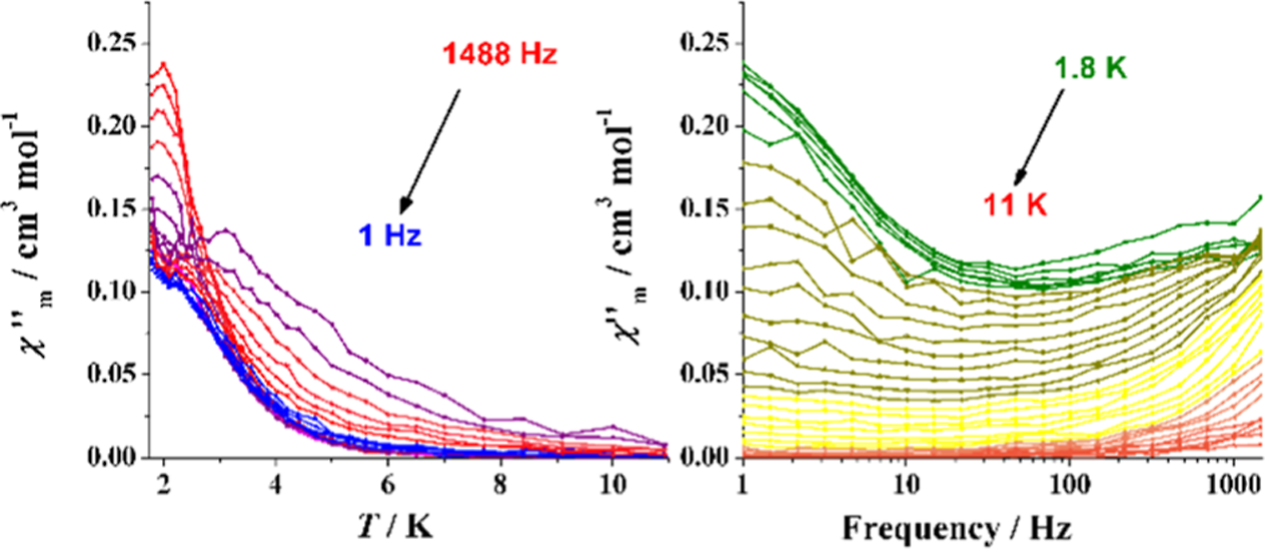
Out-of-phase response vs temperature (left) and frequency (right) for complex **1** under external field of 0.5 T.

**Figure 9:**
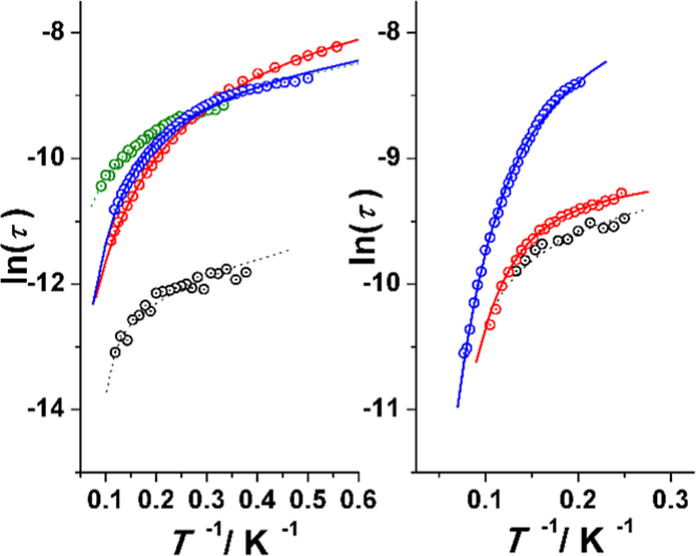
Temperature dependence of the relaxation time as a function of the temperature plotted as ln(τ) vs inverse of *T*. (Left) Complexes **1** (black), **2** (green), **3** (blue), and **4** (red). (Right) Complex **5**—0.15 T (black), **5**—0.3 T (red) and **5**—0.5 T (blue). Solid lines show the best fit of the experimental data.

Previous χ″_M_(*H*) measurements for complex **1** at the fixed frequency of 1488 Hz, as shown in Figure S9, do not show out-of-phase response at zero field, but field-dependent signals appear under field. The χ″_M_(*T*) measurements performed under the selected field of 0.5 T show a dominant temperature-independent LFT process centered at 1.9 K and HFT signals with some maximum below 3 K are shown in Figure 8. The χ″_M_(ν) plot evidences large relaxation times for the LFT process. The relaxation times extracted from the Argand plot (Figure S10), using the generalized Debye model,^55^ were fitted with the general expression
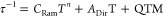
1In which the Orbach term has not been included. The best fit was obtained with the usual sum of direct plus Raman processes, but the short range of points and its dispersion yields in a large uncertainty on the *n* parameter, and thus, no fit data are reported, as shown in Figure 9, left.

**Figure 10:**
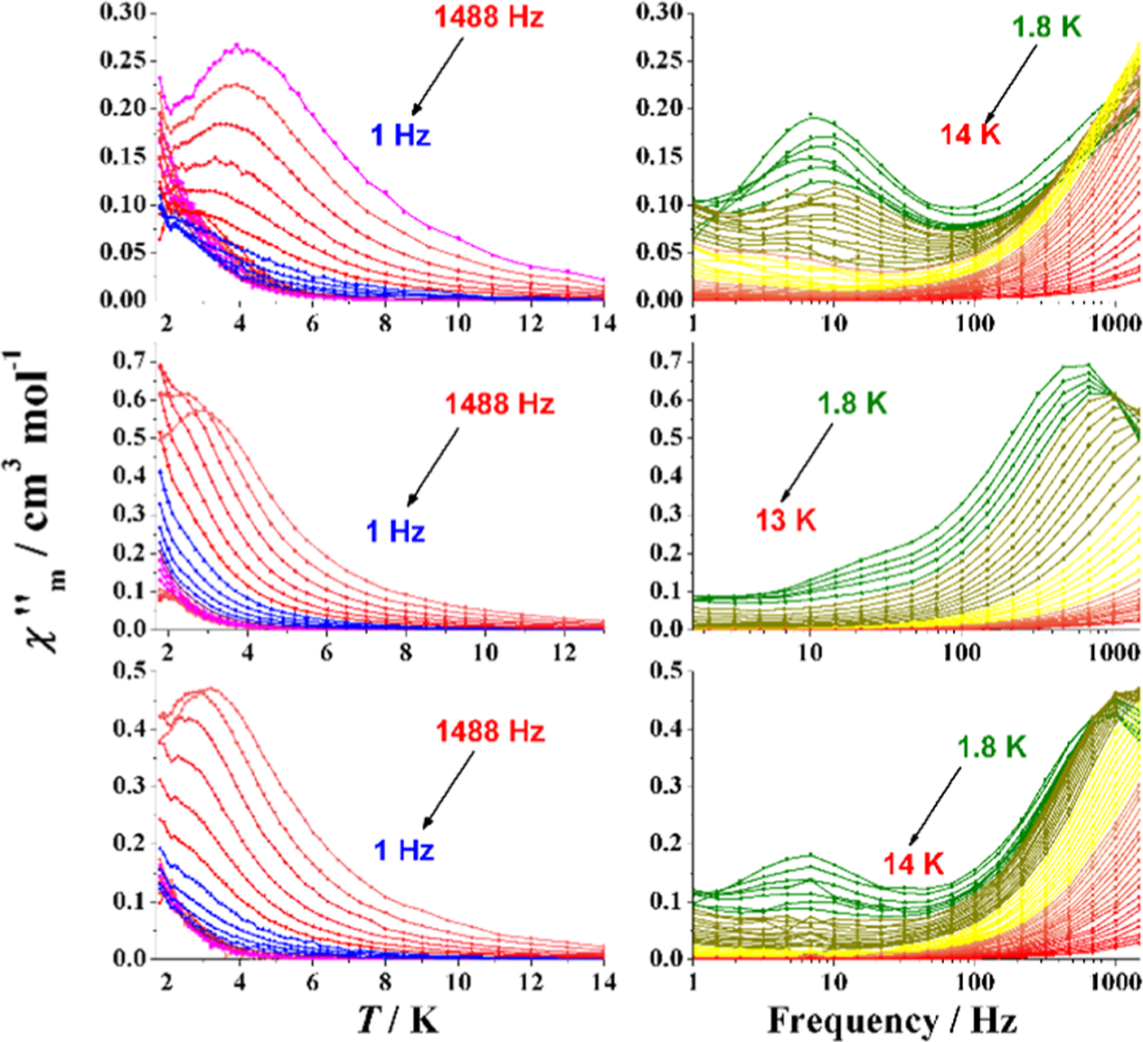
Out-of-phase response vs temperature (left) and frequency (right) for complexes **2** (Top), **3**SS (middle), and **4**SS (bottom).

As can be expected from the structural data, the ac responses of complexes **2–4** are quite similar. χ″_M_(*H*) measurements show the absence of an out-of-phase response at zero field but clear field-dependent signals, as shown in Figure S9. χ″_M_(*T*) measurements are dominated by frequency-dependent HFT signals below 5 K and tails of LFT signals close to the lower investigated temperature of 1.8 K (Figure 10). The χ″_M_(ν) plots show clear frequency-independent LFT signals centered at 10 Hz (**2**) and 8 Hz (**4**) and thus τ values ≈2 × 10^–2^ s.

The relaxation times were extracted from the Argand plots (Figure S10), in the temperature range 3–11 K for **2**, 1.8–9.0 K for **3,** and 2–8.5 K for **4**. The fit of ln(τ) vs inverse of temperature with eq 1 yields common direct plus Raman processes and, as could be expected from the similar structural data, they exhibit a similar response, as shown in Figure 9, left. Similar to complex **1**, the short range of data for **2** was poorly reliable, giving too large uncertainty on the *n* parameter and thus, the fit parameters results are reported for **3**SS and **4**SS, as shown in Table 1.

**Table 1:** Best Fit Parameters for the ln(τ) vs Inverse of Temperature for Complexes **3**SS, **4**SS, and **5**

	*C*	*n*	*A*	QTM
**3**SS	19(1)	3.8(1)	2211(79)	
**4**SS	5.5(2)	4.0(2)	2756(45)	
**5** (0.3 T)	1.0(1)	4.0(8)	711(152)	7745(635)
**5** (0.5 T)	0.35(1)	4.3(2)	606(54)	860(290)

### Static Magnetic Properties

#### Complex (μ_1,1_-N_3_)_2_[Ni_2_Mn_2_(L1)_2_(N_3_)_2_] (**5**)

**Figure 11:**
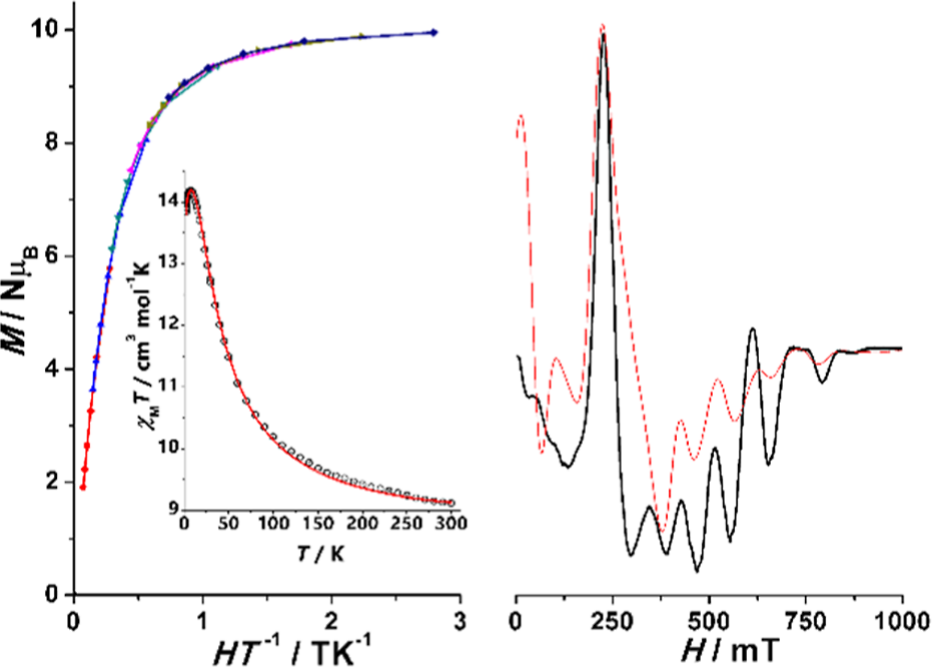
(Left) Reduced magnetization and χ_M_*T* product vs temperature for complex **5**. (Right) X-band EPR spectrum. Red lines show the best fits of the experimental data.

In agreement with previous data,^36^ applying the Hamiltonian H = −2*J*(*S*_1_·*S*_2_) complex **5** shows a ferromagnetic interaction of *J* = +2.54(1) cm^–1^ mediated by the end-on azido bridges between the manganese cations, also in good agreement with the usual values of *J* for Mn^II^–N–Mn^II^ bond angles around 100°,^52^ as shown in Figure 11-inset, resulting a well isolated ground state *S* = 5, which is placed 25.4 cm^–1^ below the first excited state *S* = 4 and, thus, fully populated at the working temperatures. Its weak anisotropy cannot be calculated from the superimposable reduced magnetization measurements (Figure 11, left) but that can be equally evaluated as *D* = ± 0.060 cm^–1^ and E/D = 0.31 from its *X*-band EPR spectrum (Figure 11-rigth), and as in the above cases, discrimination of the sign of *D* was not possible from the EPR spectrum and it was determined from theoretical calculations (see the Computational Data section). The weak anisotropy is the result of the strongly distorted environment that according to our proposal lies in the optimal *D* range to induce SRM. To verify our hypothesis, dynamic measurements were performed on **5**.

### Dynamic Magnetic Properties

#### Complex (μ_1,1_-N_3_)_2_[Ni_2_Mn_2_(L1)_2_(N_3_)_2_] (**5**)

**Figure 12:**
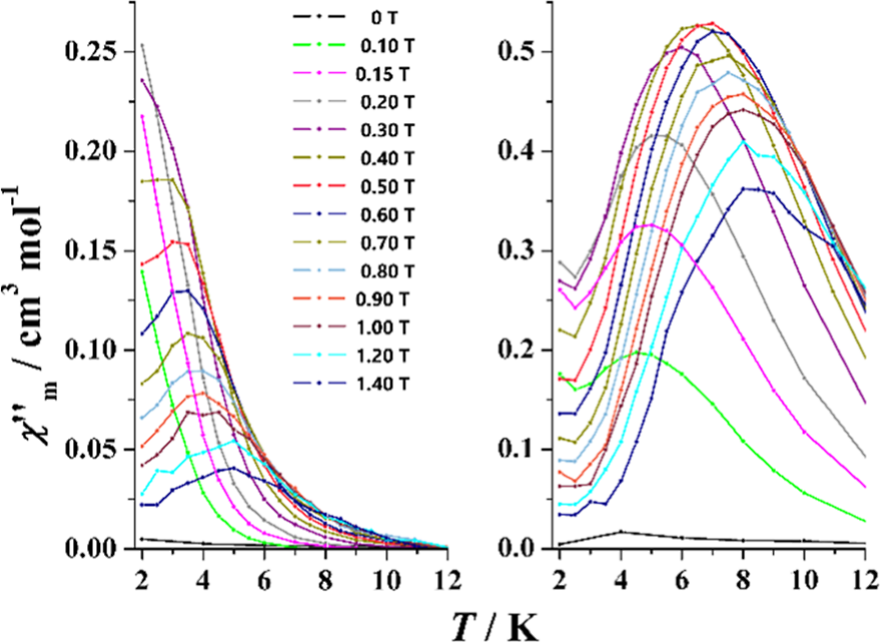
Field dependence of the out-of-phase response at 10 Hz (top) and 1000 Hz (bottom) for complex **5**. Bold lines correspond to the selected fields for the χ″_M_(*T*) measurements.

Complex **5** was initially measured at two fixed frequencies as a function of magnetic field up to a moderately strong field of 1.4 T. The LFT signals measured at 10 Hz were well-defined, and their field dependence shows a displacement to higher temperature and a fast decrease of intensity for increasing fields; in contrast, the HFT signals show a fast increase of intensity and a continuous displacement to higher temperature for increasing fields and a slow and continuous decrease of intensity above 0.5 T, as shown in Figure 12.

**Figure 13:**
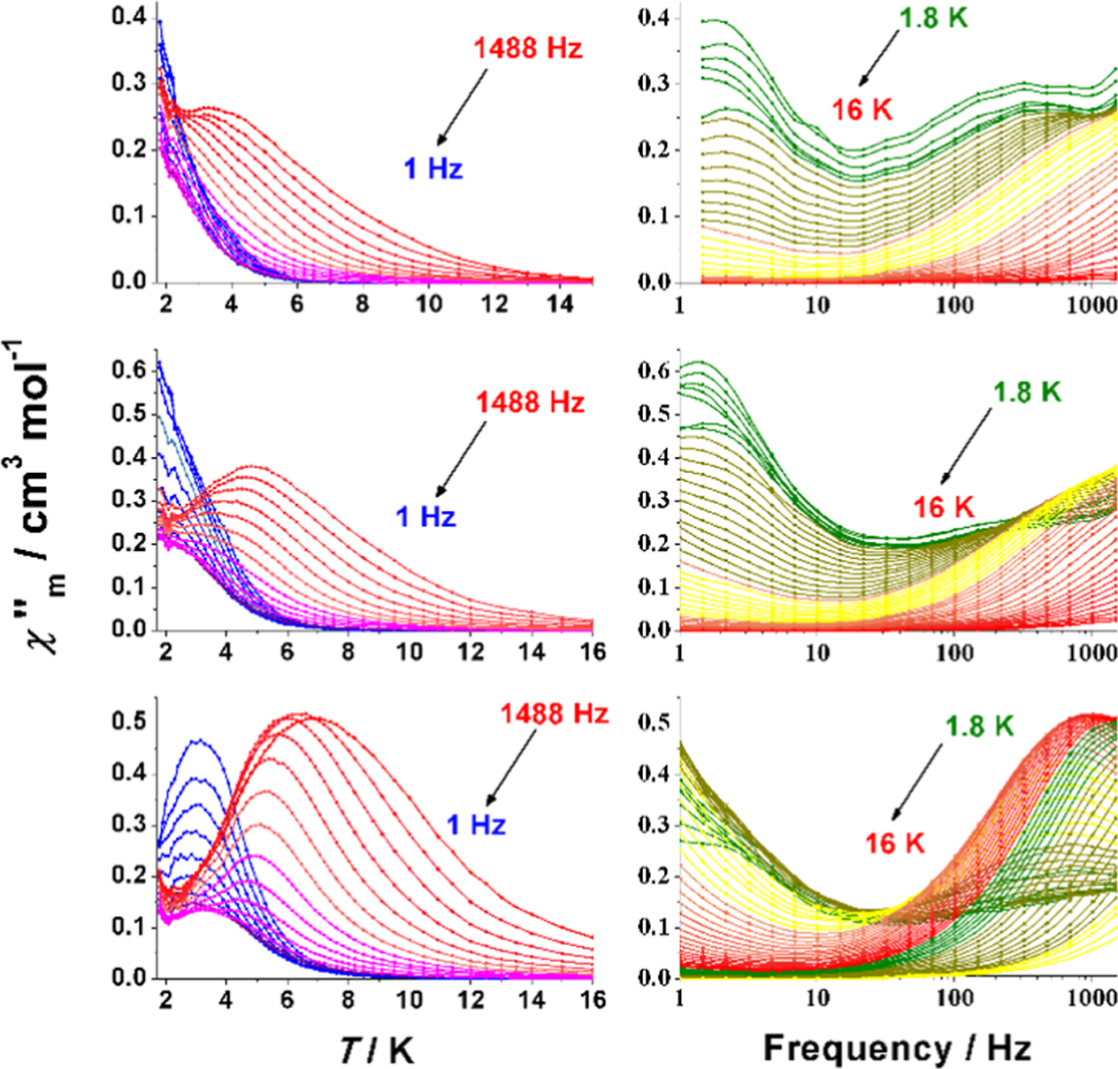
Out-of-phase response vs temperature (left) and frequency (right) for complex **5** measured under a field of 0.15 T (top), 0.3 T (middle), and 0.5 T (bottom).

In light of the good resolution of the ac response, the dependence of the out-of-phase signal as a function of the frequency (1–1485 Hz) was determined for three selected fields, 0.15, 0.30, and 0.50 T, as shown in Figure 13. The χ″_M_(*T*) plots show the shift to higher temperature of the signals for increasing field and the change in the relative intensity among the LFT and HFT signals. As in the previously reported cases, the χ″_M_(ν) plots show frequency-independent LFT signals around 2 Hz for the measurement at the low field (τ ≈ 0.08 s), 1.4 Hz for the measurement at o.3 T (τ ≈ 0.11 s), and the maxima below 1 Hz for the measurement at 0.5 T (τ > 0.15 s). For the HFT signals, the plots evidence an enlargement of the relaxation time when increasing the applied magnetic field.

Fit of the Argand plots (Figure S11) for the HFT signals was determined in the range of temperatures 4–7.5 K for 0.15 T, 4–9.5 K for 0.30 T, and 4.95–13 K for 0.50 T to avoid the overlap between both groups of signals, and the fits of the ln(τ) vs inverse of temperature show a direct plus Raman with a tunneling participation relaxation processes. Best fitting parameters are reported in Table 1.

### Computational Data

In order to understand the relative stability of the different electronic configurations and states, which can explain the observed magnetic susceptibility behavior of these manganese(II) complexes, a multiconfigurational *n*-electron valence state perturbation theory (NEVPT2) calculation was carried out. In all calculations, each manganese center presents five unpaired electrons centered in d orbitals, resulting in a spin state of S = 5/2. Supporting Information contains the input files (File S1). The computed dependence of the χ_M_*T* product at the NEVPT2 level perfectly agrees with the experimental values, reaching a value of 4.386 over a wide temperature range for all calculated compounds. Moreover, the magnetization curves increase with the magnetic field up to 4.93 Bohr magnetons.

For compound **1**, we initially considered the central binuclear {MnNi} framework (1*) by obtaining the zero-field splitting parameter as *D* = +0.077 cm^–1^ (and *E*/*D* = 0.33, indicating three equally spaced doublets). Nevertheless, in the complete molecular structure, including the two extra neutral [NiL1] fragments bounded by hydrogen bonds, *D* becomes negative equal to −0.090 cm^–1^ and *E*/*D* = 0.26, decreasing the rhombicity to improve accuracy, being close to the experimental spectroscopic values.

It is worth noting that the *D* and *E*/*D* ratio are poorly sensitive to the calculation performed on the central {MnNi} dinuclear framework 1* or the complete molecule **1,** but for the latter, it gives an opposite sign, pointing out the noninnocent participation of the H-bonded {NiL1} fragments across axial water ligands.

The calculation of the *D* parameter for the other compounds also resulted in negative values. Considering the two different molecules of the compound **4** having the Ni(μ-O)2Mn(μ-O)2Ni core, *D* takes a negative value of −0.053 and −0.056 cm^–1^ comparable to the experimental one, although slightly underestimated. Equally, the compound **5**, having a Mn_2_Ni_2_ core, gives *D* = −0.080 cm^–1^, which closely reproduces the experimental value and rhombicity, as shown in Table 2.

**Table 2:** Relative Energies (in cm^–1^) for the Zero-Field Splitting of the Sextet Ground State, Together with Its Splitting Parameters and Calculated *g* Values^a^

complex	1*	1	4	5
***S***_**0**_, ***S***_**1**_	0.00	0.00	0.00/0.00	0.00
***S***_**2**_, ***S***_**3**_	0.27	0.33	0.21/0.11	0.18
***S***_**4**_, ***S***_**5**_	0.54	0.61	0.32/0.16	0.41
*D*	0.077	–0.090	–0.053/–0.056	–0.080
*E*/*D*	0.332	0.265	0.101/0.036	0.277
***g***_**1**_	2.00207	2.00206	2.00209	2.00205
***g***_**2**_	2.00206	2.00206	2.00209	2.00206
***g***_**3**_	2.00205	2.00205	2.00209	2.00206
***g***_**iso**_	2.00206	2.00206	2.00209	2.00206

a Notes: complex 1* contains only the simplified moiety Mn(μ-O)_2_Ni, while **1** includes the complete molecule. Two data are given for complex **4**, with experimental Ni–Mn distances of 3.197 and 3.173 Å respectively.

To understand the contribution of these values, a more detailed analysis of the electronic structure is performed. Since the sexted is the only possibility for the electronic ground state, it is full weighted. Other states, such as the quadruplets, have an energy above 20,000 or 25,000 cm^–1^ before spin–orbital coupling, so they do not contribute significantly to the electronic structure and should avoid electronic transition between d orbitals. Consequently, zero-field splitting in this stretched sample generates three electronic levels with practically the same energy in a range of less than 0.6 cm^–1^, giving rise to equal populations and yielding poor anisotropy. It is also reflected in the three components for calculated *g*, which give practically identical values for all systems (closer to 2.0021). The axes of these vectors are represented in Figure S12.

The ab initio ligand field theory allows us to extract the distribution of the d orbitals. For example, it is interesting to analyze the splitting of the d-orbitals in compound **1** having a coordination geometry about the pentagonal bipyramidal coordination environment around the manganese atom. For the complete molecule, the *xz* and *yz* orbitals are the most stable, having π character out of the plane of the equatorial and axial ligands, followed by the *x*^2^–*y*^2^ and *xy* orbitals localized in the equatorial plane (although the latter two can be interchanged in the simplified model). These four orbitals are distributed in a range of 3400 cm^–1^, while the *z*^2^ orbital directed to axial water ligands is very high in energy, at 8800 cm^–1^ from the former (with a gap of 5400 cm^–1^).

However, a very different distribution is found for **4**, all within 4400 cm^–1^. The *x*^2^–*y*^2^ and *xy* orbitals are now the most stable, both having δ disposition toward the metal–metal axis, followed of the *z*^2^ orbital directed to the ring center, and finally the *xz* and *yz* orbitals interacting to terminal methoxo groups. For compound **5**, a distribution analogous to the **1** one is recovered, with 4 orbitals close in energy (at 2100 cm^–1^), and the fifth one separated at 5500 cm^–1^. This is the *xz* orbital, which is localized in the plane of the ring and interacts with both terminal and bridging azide ligands.

### Discussion of the Results

Slow relaxation of the magnetization for the Mn^II^ cation has been observed in few compounds in recent years, and the origin and determinant factors that promote this magnetic response still remain unclear or at least controversial. With the aim to clarify some of these factors, we have performed a global analysis of the reported compounds to point out the relevant ones.

**Figure 14:**
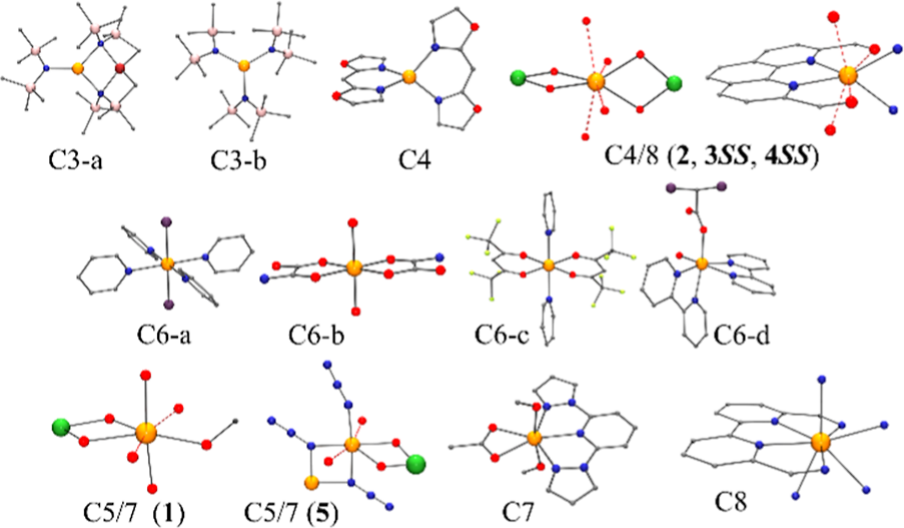
Coordination environment for the Mn^II^ complexes exhibiting SRM reported until now. For some large ligands, atoms not involved in the coordination sphere have been suppressed. Cn notation refers to the coordination number around the Mn^II^ cation. Color code: Mn^II^, orange; Ni^II^, green; O, red; N, blue; Cl, violet; Si, pink; C, gray.

In the literature, there are eight complexes exhibiting this property, for which the coordination environment has been plotted in Figure 14, together with the five complexes reported in the current work.

From Figure 14, we can realize how the coordination sphere for the Mn^II^ cations covers the complete range from tricoordination for complexes [LiMn{N(SiMe_3_)_2_}_3_] (**C3a**) and [Li(15-crown-5)][Mn{N(SiMe_3_)_2_}_3_] (**C3b**) until pseudo-octacoordination for complex **4**SS. In all the cases, the coordination polyhedron shows severe distortions derived from the bite of the bidentate ligands and their different ligand fields, which promote rhombic environments for **C6d**, **C7,** and for the complexes **1** and **5** reported in this work, or axiality for **C4**, **C6a**,**b**,**c,** and complexes **2**, **3**SS and **4**SS reported in this work. The low symmetry seems to be a determinant as the origin of some degree of anisotropy, and the SRM response is clearly nonrelated with a particular environment.

The Mn^II^ cation is isotropic with zero or quasi negligible anisotropy in comparison with other d cations such Mn^III^, Co^II^ or Ni^II^, and consequently, it has been excluded from the slow magnetic relaxation in molecules research field, in which a high *D* value has been thought to be crucial. However, it seems clear now that some degree of anisotropy is required to reach the SRM in this kind of systems and its evaluation is a critical point. The reported *D* values and the *E*/*D* ratio for the complexes plotted in Figure 14, evaluated from magnetometry, theoretical calculations or EPR spectroscopy, have been summarized in Table 3. EPR spectroscopy provides the most reliable data, because the spectra are very sensitive to even small changes in the *D* or *E* values.

**Table 3:** *D* and *D*/*E* Values for the Mn^II^ Complexes Plotted in Figure 15

complex	donors	*D* (cm^1^)	*E*/*D*	ref
**C3–a**	N_3_	0.23^[^a^]^	0.44	(23)
**C3–b**	N_3_	–0.48^[^a^]^	0.42	(23)
**C4**	N_4_	0.5^[^a^]^	0	(22)
**C4/8**(**4**SS)	O_8_	–0.053^[^b^]^	0.10	t.w
		|0.205|^[^d^]^	0.036	
**C4/8**	O_4_N_4_	–0.065^[^c^]^	0.057	(25)
**C6–a**	Cl_2_N_4_	–0.63^[^a^]^	N.R.	(19)
		–1.49^[^b^]^	N.R.	
		0.07^[^c^]^	0.05	
		0.15^[^d^]^	N.R.	
**C6–b**	O_6_	N.R.	N.R.	(21)
**C6–c**	N_2_O_4_	0.84^[^a^]^	N.R.	(24)
		0.030^[^b^]^	0.08	
		0.0784^[^d^]^	0.24	
**C6–d**	N_4_O_2_	N.R.	N.R.	(18)
**C7**	N_3_O_4_	0.491^[^a^]^	9.2 × 10^–4^	(20)
		–0.423^[^a^]^	0 (fixed)	
		–0.13^[^d^]^	0.30	
**C5/7**(**1**)	O_5_/O_7_	–0.090^[^b^]^	0.26	t.w
		|0.080|^[^d^]^	0.20	
**C5/7**(**5**)	N_3_O_2/4_	0.080^[^b^]^	0.28	t.w
		|0.060|^[^d^]^	0.31	
**C8**	N_8_	–0.026^[^c^]^	0.035	(25)

a From χ_M_*T* and/or reduced *M* data.

b From DFT calculations.

c From CASSCF calculations.

d From EPR spectrum. This work: t.w.

Boca^19,24^ and Yamashita^20^ provided comparative values and inspection of the data for complexes **C6a**, **C6c,** and **C7**, which evidence that the *D* values calculated from magnetometry are strongly overestimated. The reason for this is that it is not possible to obtain reliable *D* values from superimposable reduced magnetization plots and that the small decay at low temperature in the χ_M_*T* plots can be due to the correlated *D* and z*J*′ parameters and thus, if intermolecular interactions are not considered, *D* becomes overestimated. An additional indication is provided by the deep study performed by Duboc,^29,30^ for penta- and hexa-coordinated systems with N, O–donors, for which *D* values lower than 0.2 cm^–1^ must be expected in excellent agreement with the *D* values calculated from EPR for **C6a**, **C6c**, **C7**, and complexes **1**, **4**SS, and **5**. Accurate theoretical calculations provide a better approach than magnetometry to evaluate the magnitude of *D* and clearly give the best indication for its sign and its degree of axiality, parametrized as E/D, but for this small order of magnitude, the calculated values are still relatively far from the more precise EPR data, as was also suggested by Yamashita.^20^

**Figure 15:**
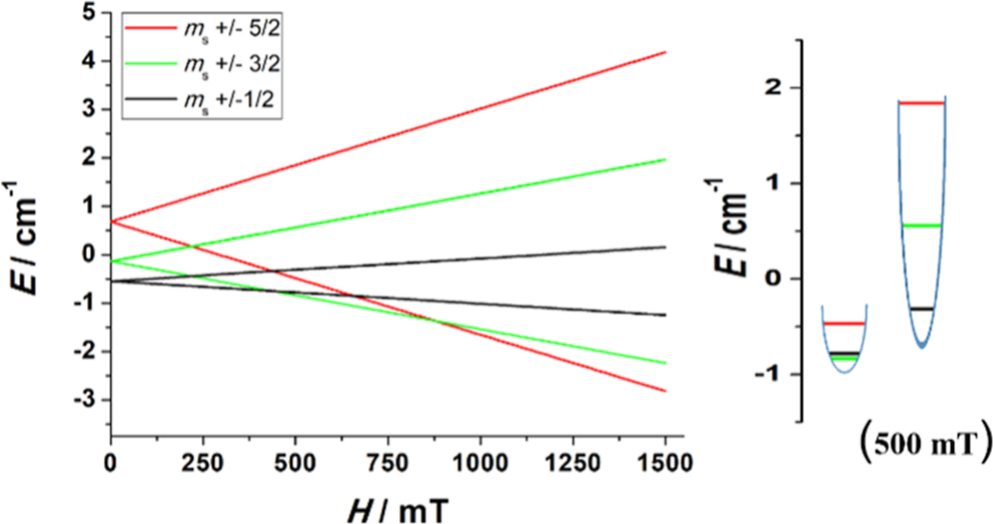
(Left) Example of Zeeman splitting for the 5/2 spin with the parameters *D* = 0.205 cm^–1^ and *E*/*D* = 0.036 found for complex **4**SS. (Right) “Double-well” under a field of 500 mT.

The almost temperature-independent LFT relaxation channel that appear in some cases^18,20,24^ has been suggested to follow a direct process in the vast majority of cases. The temperature-dependent HFT channel usually gives a linear ln(1/2πν) vs *T*^–1^ dependence with apparent high *U*_eff_ that cannot be related with an Orbach process because the calculated energies are larger than the Zeeman splitting derived from low *D* values that only reach 1–2 cm^–1^ under static fields lower than 1 T usually employed in the measurements. These low *D* values have as a consequence the crossing of *m*_s_ levels for moderate fields, as can be seen, for example, in the Zeeman plot built employing the *D* and *E*/*D* values from the EPR data for complex **4**SS, as shown in Figure 15.

This plot evidences that Orbach relaxation is not possible and that for large fields, the system tends to a Zeeman splitting close to an isotropic cation. The crossing of *m*_s_ levels provides a relaxation path that is not operative for high fields, in agreement with the decrease of the intensity of the χ_M_″ signal that vanishes for high fields.

Fit of the relaxation times has been performed as direct or combined direct plus Raman processes in all cases,^18−24^ excluding the Orbach process. The values of the *n* coefficient have been related with the relaxation process, with *n* = 2 indicative of phonon bottleneck process and *n* = 1 or *n* > 7 for the pure direct or Raman processes.^56^ The lower reported *n* value of 1.09 was found^20^ for the heptacoordinate complex **C7,** whereas for the remaining cases, values close or slightly larger than 2 were reported, suggesting a typical phonon bottleneck or some degree of mixing of two processes, as happens also in the systems reported in this work.

## Conclusions

The series of complexes obtained from the cascade reaction of compartmental Schiff bases with Ni^II^ and Mn^II^ yielded several complexes with the diamagnetic nickel cation in a square planar environment with {Ni_3_Mn}, {Ni_2_Mn}, and {Mn_2_} nuclearities. From EPR measurements, low *D* values in the 10^–1^–10^–2^ cm^–1^ order of magnitude were found, and the negative sign was determined from NEVPT2 calculations. In all cases, the reported complexes exhibit slow relaxation of the magnetization, and, from comparison with previously reported systems, it can be concluded that (a) a low *D* value becomes crucial to promote SRM in this kind of isotropic systems, (b) sign of *D* or rhombic/axial distortion is not determinant to promote SRM in the Mn^II^ case; (c) for the first time, it has been proved that polynuclear high spin Mn^II^ complexes with large ground *S* levels are also able to exhibit SRM.

Further experiments, with such larger variations of *D*, systematic field dependence analysis or larger nuclearities, and *S* ground state are desirable to fully characterize this unusual property.

## Supporting Information Available

Additional structural, magnetic data, and ORCA inputs (PDF)

## References

[ref1] Sessoli R.; Gatteschi D.; Caneschi A.; Novak M. A. Magnetic bistability in a metal-ion cluster. Nature 1993, 365, 141–143. 10.1038/365141a0.

[ref2] Gatteschi D.; Sessoli R.; Villain J. Molecular Nanomagnets; Oxford University Press, 2006.

[ref3] Frost J. M.; Harriman K. L. M.; Murugesu M. The rise of 3-d single-ion magnets in molecular magnetism: towards materials from molecules?. Chem. Sci. 2016, 7, 2470–2491. 10.1039/C5SC03224E. 28660017 PMC5477015

[ref4] Maniaki D.; Pilichos E.; Perlepes S. P. Coordination clusters of 3d-metals that behave as single-molecule magnets (SMMs): synthetic routes and strategies. Front. Chem. 2018, 6, 461 10.3389/fchem.2018.00461. 30356793 PMC6190736

[ref5] Coronado E. Molecular magnetism: from chemical design to spin control in molecules, materials and devices. Nat. Rev. Mater. 2020, 5, 87–104. 10.1038/s41578-019-0146-8.

[ref6] Bogani L.; Wernsdorfer W. Molecular spintronics using single-molecule magnets. Nat. Mater. 2008, 7, 179–186. 10.1038/nmat2133. 18297126

[ref7] Escalera-Moreno L.; Baldovi J. J.; Gaita-Ariño A.; Coronado E. Spin states, vibrations and spin relaxation in molecular nanomagnets and spin qubits: a critical perspective. Chem. Sci. 2018, 9, 3265–3275. 10.1039/C7SC05464E. 29780458 PMC5935026

[ref8] Zabala-Lekuona A.; Seco J. M.; Colacio E. Single-molecule magnets: From Mn_12_-ac to dysprosium metallocenes, a travel in time. Coord. Chem. Rev. 2021, 441, 213984 10.1016/j.ccr.2021.213984.

[ref9] Juráková J.; Šalitroš I. Co(II) single-ion magnets: synthesis, structure, and magnetic properties. Monatsh. Chem. 2022, 153, 1001–1036. 10.1007/s00706-022-02920-0. 35615113 PMC9123880

[ref10] Tkac V.; Orendacova A.; Tarasenko R.; Cizmar E.; Orendac M.; Tibenska K.; Anders A. G.; Gao S.; Pavlık V.; Feher A. Multiple-timescale relaxation dynamics in CsGd(MoO4)2 - a dipolar magnet with a highly anisotropic layered crystal structure. J. Phys.: Condens. Matter 2013, 25, 506001 10.1088/0953-8984/25/50/506001. 24275898

[ref11] Girginova P. I.; Pereira L. C. J.; Coutinho J. T.; Santos I. C.; Almeida M. Slow magnetic relaxation in lanthanide ladder type coordination polymers. Dalton Trans. 2014, 43, 1897–1905. 10.1039/C3DT52748D. 24264729

[ref12] Arauzo A.; Lazarescu A.; Shova S.; Bartolomé E.; Cases R.; Luzón J.; Bartolomé J.; Turta C. Structural and magnetic properties of some lanthanide (Ln = Eu(III), Gd(III) and Nd(III)) cyanoacetate polymers: field-induced slow magnetic relaxation in the Gd and Nd substitutions. Dalton Trans. 2014, 43, 12342–12356. 10.1039/C4DT01104J. 24988294

[ref13] Calahorro A. J.; Oyarzabal I.; Fernández B.; Seco J. M.; Tian T.; Fairen-Jimenez D.; Colacio E.; Rodríguez-Diéguez A. Rare earth anthracenedicarboxylate metal-organic frameworks: slow relaxation of magnetization of Nd^3+^, Gd^3+^, Dy^3+^, Er^3+^ and Yb^3+^ based materials. Dalton Trans. 2016, 45, 591–598. 10.1039/C5DT03946K. 26610692

[ref14] Izuogu D. C.; Yoshida T.; Zhang H.; Cosquer G.; Katoh K.; Ogata S.; Hasegawa M.; Nojiri H.; Damjanovic M.; Wernsdorfer W.; Uruga T.; Ina T.; Breedlove B. K.; Yamashita M. Slow magnetic relaxation in a palladium–gadolinium complex induced by electron density donation from the palladium ion. Chem.—Eur. J. 2018, 24, 9169–9294. 10.1002/chem.201802702. 29663534

[ref15] Vráblová A.; Tomás M.; Falvello L. R.; Dlháň Ĺ.; Titiš J.; Černák J.; Boča R. Slow magnetic relaxation in Ni–Ln (Ln = Ce, Gd, Dy) dinuclear complexes. Dalton Trans. 2019, 48, 13943–13952. 10.1039/C9DT02122A. 31441924

[ref16] Mayans J.; Escuer A. Correlating the axial zero field splitting with the slow magnetic relaxation in Gd^III^ SIMs. Chem. Commun. 2021, 57, 721–724. 10.1039/D0CC07474H. 33496705

[ref17] Mylonas-Margaritis I.; Lada Z. G.; Kitos A. A.; Maniaki D.; Skordi K.; Tasiopoulos A. J.; Bekiari V.; Escuer A.; Mayans J.; Nastopoulos V.; Bakalbassis E. G.; Papaioannou D.; Perlepes S. P. Interesting chemical and physical features of the products of the reactions between trivalent lanthanoids and a tetradentate Schiff base derived from cyclohexane-1,2-diamine. Dalton Trans. 2023, 52, 8332–8343. 10.1039/D3DT00817G. 37259668

[ref18] Benniston A. C.; Melnic S.; Turta C.; Arauzo A. B.; Bartolomé J.; Bartolomé E.; Harrington R. W.; Probert M. R. Preparation and properties of a calcium(II)-based molecular chain decorated with manganese(II) butterfly-like complexes. Dalton Trans. 2014, 43, 13349–13357. 10.1039/C4DT01518E. 25078125

[ref19] Rajnak C.; Titis J.; Moncol J.; Micova R.; Boca R. Field-induced slow magnetic relaxation in a mononuclear manganese(II) complex. Inorg. Chem. 2019, 58, 991–994. 10.1021/acs.inorgchem.8b02675. 30624897

[ref20] Uchida K.; Cosquer G.; Sugisaki K.; Matsuoka H.; Sato K.; Breedlove B. K.; Yamashita M. Isostructural M(II) complexes (M = Mn, Fe, Co) with field-induced slow magnetic relaxation for Mn and Co complexes. Dalton Trans. 2019, 48, 12023–12030. 10.1039/C8DT02150C. 31298228

[ref21] DaCunha T. T.; Barbosa V. M. M.; Oliveira W. X. C.; Pedroso E. F.; Garcıa D. M. A.; Nunes W. C.; Pereira C. L. M. Field-induced slow magnetic relaxation of a six-coordinate mononuclear manganese(II) and cobalt(II) oxamate complexes. Inorg. Chem. 2020, 59, 12983–12987. 10.1021/acs.inorgchem.0c01628. 32897061

[ref22] Legendre C. M.; Luert D.; Herbst-Irmer R.; Stalke D. Benchmarking magnetic and spectroscopic properties on highly stable 3d metal complexes with tuneable bis(benzoxazol-2-yl)methanide ligands. Dalton Trans. 2021, 50, 16810–16818. 10.1039/d1dt03230e. 34766963

[ref23] Indris S.; Bredow T.; Schwarz B.; Eichhöfer A. Paramagnetic ^7^Li NMR shifts and magnetic properties of divalent transition metal silylamide ate complexes [LiM{N(SiMe_3_)_2_}_3_] (M^2+^ = Mn, Fe, Co). Inorg. Chem. 2022, 61, 554–567. 10.1021/acs.inorgchem.1c03237. 34931842

[ref24] Micova R.; Rajnak C.; Titis J.; Samolova E.; Zalibera M.; Bienko A.; Boca R. Slow magnetic relaxation in two mononuclear Mn(II) complexes not governed by the over-barrier Orbach process. Chem. Commun. 2023, 59, 2612–2615. 10.1039/D2CC06510J. 36757181

[ref25] Wang L. X.; Wu X. F.; Jin X. X.; Li J. Y.; Wang B. W.; Liu J. Y.; Xiang J.; Gao S. Slow magnetic relaxation in 8-coordinate Mn(II) compounds. Dalton Trans. 2023, 52, 14797–14806. 10.1039/D3DT02307A. 37812439

[ref26] Mayans J.; Font-Bardia M.; Escuer A. Chiroptical and magnetic properties of star-shaped Fe^III^_4_ complexes from chiral Schiff bases. Structural and magnetic correlations based on continuous shape measures. Dalton Trans. 2018, 47, 8392–8401. 10.1039/C8DT01684D. 29897079

[ref27] Aravena D.; Venegas-Yazigi D.; Ruiz E. Single-molecule magnet properties of transition-metal ions encapsulated in lacunary polyoxometalates: a theoretical study. Inorg. Chem. 2016, 55, 6405–6413. 10.1021/acs.inorgchem.6b00145. 27299178

[ref28] Wang J.; Jing Y.; Cui M.; Lu Y. M.; Ouyang Z.; Shao C.; Wang Z.; Song Y. Spin Qubit in a 2D Gd^III^Na^I^-based oxamato supramolecular coordination framework. Chem.—Eur. J. 2023, 29, e202301771 10.1002/chem.202301771. 37665775

[ref29] Duboc C.; Phoeung T.; Zein S.; Pecaut J.; Collomb M. N.; Neese F. Origin of the Zero-Field Splitting in mononuclear octahedral dihalide Mn^II^ complexes: an investigation by multifrequency high-field electron paramagnetic resonance and density functional theory. Inorg. Chem. 2007, 46, 4905–4916. 10.1021/ic062384l. 17508742

[ref30] Duboc C. Determination and prediction of the magnetic anisotropy of Mn ions. Chem. Soc. Rev. 2016, 45, 5834–5847. 10.1039/C5CS00898K. 27508279

[ref31] Ghosh T. K.; Maity S.; Mayans J.; Ghosh A. Family of isomeric Cu^II^–Ln^III^ (Ln = Gd, Tb, and Dy) complexes presenting field-induced slow relaxation of magnetization only for the members containing Gd^III^. Inorg. Chem. 2021, 60, 438–448. 10.1021/acs.inorgchem.0c03129. 33351616

[ref32] Caballero S.; Pilichos E.; Font-Bardia M.; Mayans J.; Escuer A. Field-induced slow magnetic relaxation in a new family of tetranuclear double-stranded Cu_2_^II^–Ln_2_^III^ metallohelicates. Cryst. Growth Des. 2023, 23, 3711–3719. 10.1021/acs.cgd.3c00121.

[ref33] Pilichos E.; Bhunia P.; Font-Bardia M.; Ghosh A.; Mayans J.; Escuer A. Quasi-isotropic SMMs: slow relaxation of the magnetization in polynuclear Cu^II^/Mn^II^ complexes. Dalton Trans. 2022, 51, 1779–1783. 10.1039/D1DT04074J. 35076050

[ref34] Pilichos E.; Font-Bardia M.; Escuer A.; Mayans J. Occurrence of slow relaxation of the magnetization in a family of copper(II)/manganese(II) quasi-isotropic complexes with different ground spin states. Dalton Trans. 2022, 51, 17653–17663. 10.1039/D2DT02807G. 36342234

[ref35] Shcherbakov I. N.; Krotkii I. I.; Kazachkova V. I.; Lyubchenko S. N.; Efimov N. N.; Tsaturyan A. A.; Lazarenko V. A. Field induced slow magnetic relaxation in a linear homotrinuclear manganese heterospin coordination compound with S = 7/2 ground state and intriguing spin density distribution. Dalton Trans. 2024, 53, 6860–6864. 10.1039/D3DT04123A. 38584467

[ref36] Jia H. P.; Li W.; Ju Z. F.; Zhang J. Synthesis, structure, and magnetic properties of a novel mixed-bridged heterometal tetranuclear complex [Mn_2_Ni_2_(MeOSalen)_2_(μ_1,1_-N_3_)_2_(N_3_)_2_]. Inorg. Chem. Commun. 2007, 10, 397–400. 10.1016/j.inoche.2006.12.009.

[ref37] Bain G. A.; Berry J. F. Diamagnetic Corrections and Pascal’s Constants. J. Chem. Educ. 2008, 85, 532 10.1021/ed085p532.

[ref38] Sheldrick G. M. SHELXL-2014/7: Program for the Solution of Crystal Structures; University of Göttingen: Göttingen, Germany, 2014.

[ref39] Angeli C.; Cimiraglia R.; Evangelisti S.; Leininger T.; Malrieu J. P. Introduction of *n*-electron valence states for multireference perturbation theory. J. Chem. Phys. 2001, 114, 10252–10264. 10.1063/1.1361246.

[ref40] Angeli C.; Cimiraglia R. Multireference perturbation configuration interaction V. Third-order energy contributions in the Møller–Plesset and Epstein–Nesbet partitions. Theor. Chem. Acc. 2002, 107, 313–317. 10.1007/s00214-002-0336-z.

[ref41] Angeli C.; Cimiraglia R.; Malrieu J. P. N-electron valence state perturbation theory: a fast implementation of the strongly contracted variant. Chem. Phys. Lett. 2001, 350, 297–305. 10.1016/S0009-2614(01)01303-3.

[ref42] Neese F. Software update: The ORCA program system-Version 5.0. Wiley Interdiscip. Rev. Comput. Mol. Sci. 2022, 12, e1606 10.1002/wcms.1606.

[ref43] Jansen G.; Hess B. A. Revision of the Douglas-Kroll transformation. Phys. Rev. A 1989, 39, 6016–6017. 10.1103/PhysRevA.39.6016. 9901188

[ref44] Weigend F.; Ahlrichs R. Balanced basis sets of split valence, triple zeta valence and quadruple zeta valence quality for H to Rn: Design and assessment of accuracy. Phys. Chem. Chem. Phys. 2005, 7, 3297–3305. 10.1039/b508541a. 16240044

[ref45] Schäfer A.; Horn H.; Ahlrichs R. J. Fully optimized contracted Gaussian basis sets for atoms Li to Kr. J. Chem. Phys. 1992, 97, 2571–2577. 10.1063/1.463096.

[ref46] Schäfer A.; Huber C.; Ahlrichs R. Fully optimized contracted Gaussian basis sets of triple zeta valence quality for atoms Li to Kr. J. Chem. Phys. 1994, 100, 5829–5835. 10.1063/1.467146.

[ref47] Maurice R.; Bastardis R.; Graaf C. d.; Suaud N.; Mallah T.; Guihéry N. Universal theoretical approach to extract anisotropic spin Hamiltonians. J. Chem. Theory Comput. 2009, 5, 2977–2984. 10.1021/ct900326e. 26609979

[ref48] Atanasov M.; Aravena D.; Suturina E.; Bill E.; Maganas D.; Neese F. First principles approach to the electronic structure, magnetic anisotropy and spin relaxation in mononuclear 3d-transition metal single molecule magnets. Coord. Chem. Rev. 2015, 289–290, 177–214. 10.1016/j.ccr.2014.10.015.

[ref49] Sarkar S.; Nayak M.; Fleck M.; Dutta S.; Florke U.; Koner R.; Mohanta S. Syntheses, crystal structures and mass spectrometry of mononuclear Ni^II^ inclusion product and self-assembled [2 × 1 + 1 × 2] Ni^II^_3_M^II^ (M = Cu, Ni, Co, Fe or Mn) cocrystals derived from N,N’-ethylenebis(3-ethoxysalicylaldimine). Eur. J. Inorg. Chem. 2010, 2010, 735–743. 10.1002/ejic.200900685.

[ref50] Mayans J.; Saez Q.; Font-Bardia M.; Escuer A. Enhancement of magnetic relaxation properties with 3d diamagnetic cations in [Zn^II^Ln^III^] and [Ni^II^Ln^III^], Ln^III^ = Kramers lanthanides. Dalton Trans. 2019, 48, 641–652. 10.1039/C8DT03679A. 30540317

[ref51] Llunell M.; Casanova D.; Cirera J.; Alemany P.; Alvarez S. Shape. version 2.0: Barcelona, 2010.

[ref52] Escuer A.; Esteban J.; Perlepes S. P.; Stamatatos T. C. The bridging azido ligand as a central “player” in high-nuclearity 3d- metal cluster chemistry. Coord. Chem. Rev. 2014, 275, 87–129. 10.1016/j.ccr.2014.04.001.

[ref53] Chilton N. F.; Anderson R. P.; Turner L. D.; Soncini A.; Murray K. S. PHI: a powerful new program for the analysis of anisotropic monomeric and exchange-coupled polynuclear *d*- and *f*-block complexes. J. Comput. Chem. 2013, 34, 1164–1175. 10.1002/jcc.23234. 23386394

[ref54] Stoll S.; Schweiger A. EasySpin, a comprehensive software package for spectral simulation and analysis in EPR. J. Magn. Reson. 2006, 178, 42–55. 10.1016/j.jmr.2005.08.013. 16188474

[ref55] Cole K. S.; Cole R. H. Dispersion and absorption in dielectrics I. Alternating current characteristics. J. Chem. Phys. 1941, 9, 341–351. 10.1063/1.1750906.

[ref56] Shrivastava K. N. Theory of spin-lattice relaxation. Phys. Status Solidi 1983, 117, 437–458. 10.1002/pssb.2221170202.

