# Supplementary material for: Family of Quasi-Isotropic MnII and Mn2II Complexes Exhibiting Slow Relaxation of the Magnetization
Source: Inorg Chem. 2024 Oct 16;63(43):20415–26. doi: 10.1021/acs.inorgchem.4c02826 (PMC11523239; doi:10.1021/acs.inorgchem.4c02826)
Supplement: Supplementary file 1 — ic4c02826_si_001.pdf [file ic4c02826_si_001.pdf]

Supporting Information

A family of quasi-isotropic Mn^{II} and Mn^{II}_2 complexes exhibiting slow relaxation of the magnetization

Evangelos Pilichos[†], Mercè Font-Bardia[‡], Gabriel Aullón,[?] Júlia Mayans[†] and Albert Escuer *[†]

[†] *Departament de Química Inorgànica i Orgànica, Secció Inorgànica and Institute of Nanoscience and Nanotecnology (IN²UB), Universitat de Barcelona, Marti i Franques 1-11, Barcelona-08028, Spain.*
[‡] *Departament de Mineralogia, Cristal·lografia i Dipòsits Minerals, Universitat de Barcelona, Martí Franqués s/n, 08028 Barcelona (Spain) and Unitat de Difracció de R-X. Centre Científic i Tecnològic de la Universitat de Barcelona (CCiTUB), Solé i Sabarís 1-3. 08028 Barcelona.*
[?] *Departament de Química Inorgànica i Orgànica, Secció Inorgànica and Institut de Química Teòrica i Computacional (IQTCUB), Universitat de Barcelona, 08028 Barcelona, Spain.*
*Albert Escuer, Email: albert.escuer@qi.ub.edu

Table S1. Crystal data and refinement details for compounds **1**, **2**, **3*SS***, **4*RR*** and **4*SS***.

Table S2. Crystal data for complex **NiL3**.

Table S3. Main bond distances (Å) and bond angles (deg.) for [NiL3(H2O)] (**NiL3**).

Table S4. Selected bond distances (Å) and angles (deg.) for complex **1**.

Table S5. Parameters for the H-bonds between the neutral [NiL1] fragments and the four O-atoms of the Schiff bases for complex **1**.

Table S6. Selected bond distances (Å) and angles (deg.) for complex **2**.

Table S7. Selected bond distances (Å) and angles (deg.) for complex **3*SS***.

Table S8. Selected bond distances (Å) and angles (deg.) for complexes **4*RR*** and **4*SS***.

Table S9. Main bond (Å) and angles (deg.) for complex **5**.

Figure S1. Powder X-ray diffraction for complex **5**.

Figure S2. Partially labelled plot for [NiL3(H_2O)] (**NiL3**).

Figure S3. IR spectra for complexes **1** – **5**.

Figure S4. EDC spectra for the pairs of enantiomeric complexes **3*RR***/**3*SS*** and **4*RR***/**4*SS***.

Figure S5. Closest coordination polyhedra around the Mn^{II} cations in complexes **1** – **5**.

Figure S6. Left, a view of the unit cell along c-axis of complex **1**. Right, pillared arrangement of *sandwich* units of **1**.

Figure S7. Arrangement of the trimeric units in the network for complexes **2** (top), **3*SS*** (middle) and **4*SS*** (bottom).

Figure S8. Reduced magnetization and $\chi_M T$ plot (inset) for **2**, (left), **3*SS*** (middle) and **4*SS*** (right).

Figure S9. Field dependence of the out-of-phase response of complexes **1** - **4** measured under a fixed frequency of 1488 Hz

Figure S10. Argand plots for complexes **1** – **4**.

Figure S11. Argand plots for complex **5** under different external fields.

Figure S12. The three components for calculated g-matrix for complexes **1**, **4** and **5**.

File S1. ORCA input files for the CASSCF/NEVPT2 calculations on the reoriented experimental geometry.

Table S1. Crystal data and refinement details for compounds **1**, **2**, **3*SS***, **4*RR*** and **4*SS***.

	**1**	**2**	**3SS**	**4RR**	**4SS**
Formula	$C_{56}H_{66}Cl_2Mn N_6Ni_3O_{24}$	$C_{78}H_{86}Cl_{14}Mn_2 N_8Ni_4O_{33}$	$C_{47}H_{56}Cl_6Mn N_4Ni_2O_{17}$	$C_{60}H_{52}Cl_2Mn N_4Ni_2O_{16}$	$C_{60}H_{52}Cl_2Mn N_4Ni_2O_{16}$
FW	1509.11	2504.56	1334.01	1328.31	1328.31
System	Monoclinic	Monoclinic	Monoclinic	Orthorombic	Orthorombic
Space group	P21/c	P21/n	P21	P2221	P2221
a/Å	15.460(2)	15.4867(11)	8.4442(6)	14.4382(9)	14.4195(3)
b/Å	22.118(3)	16.9740(11)	14.2499(10)	18.2949(8)	18.2819(3)
c/Å	17.992(3)	19.4904(14)	22.4771(18)	23.1532(12)	23.0690(5)
α/deg.	90	90	90	90	90
β/deg.	95.684(5)°	93.893(3)°	95.482(3)	90	90
γ/deg.	90	90	90	90	90
V/ $Å^3$	6122(1)	5111.6(6)	2692.3(3)	6115.8(6)	6081.4(2)
Z	4	2	2	4	4
T, K	100(2)	100(2)	100(2)	100(2)	100(2)
λ(MoKα), Å	0.71073	0.71073	0.71073	0.71073	0.71073
ρcalc, g·cm^{-3}	1.637	1.627	1.646	1.443	1.451
μ(MoKα), mm^{-1}	1.285	1.408	1.295	0.970	0.976
Flack param.	---	---	0.017(6)	0.01(1)	0.00(1)
R	0.0242	0.0274	0.0541	0.0578	0.434
ωR2	0.0565	0.0741	0.1294	0.1057	0.1037

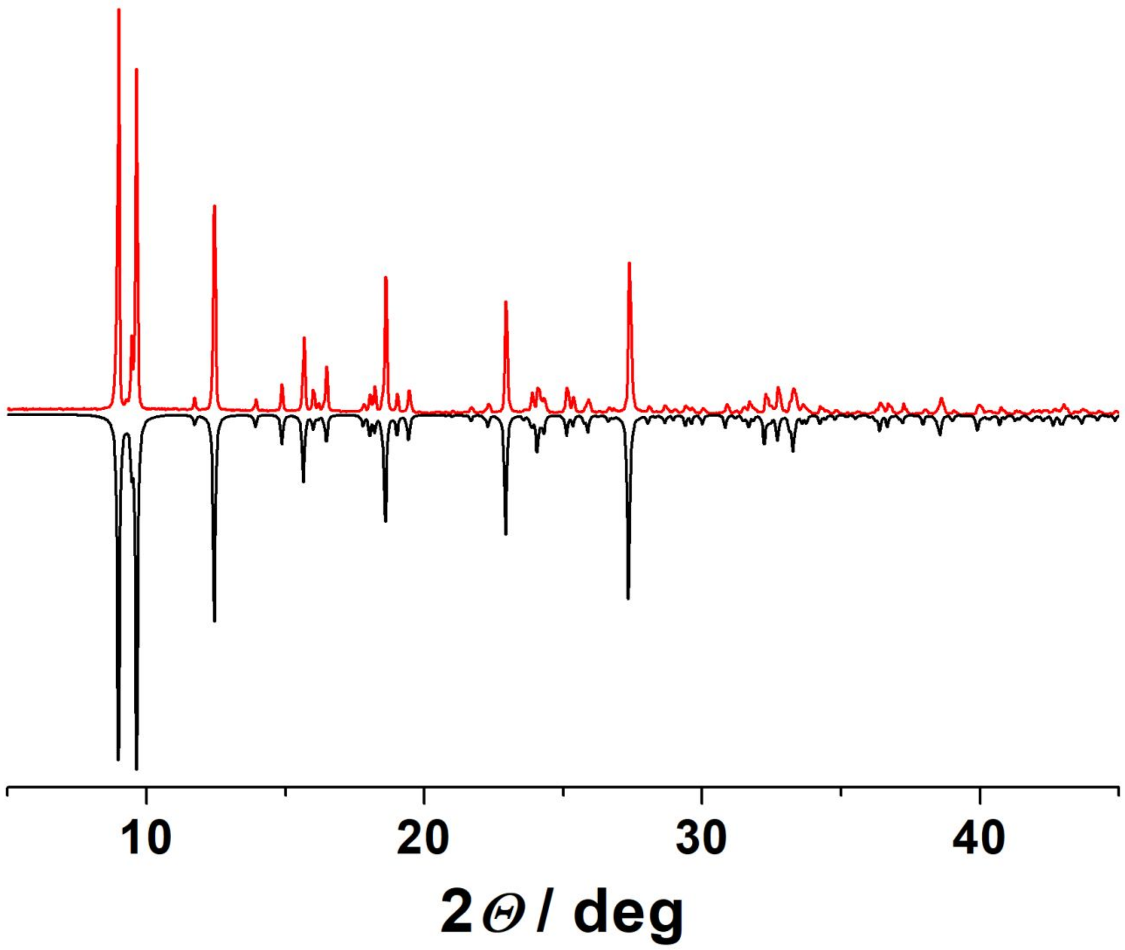

Figure S1. Powder X-ray diffraction for complex **5**. Black line, simulated spectrum from CCCD-CIBPAF; red line, experimental spectrum.

Table S2. Crystal data for complex **NiL3**.

	**NiL3**
Formula	$C_{31}H_{32}NiN_2O_6$
FW	587.29
System	Triclinic
Space group	P1
a/Å	10.3053(14)
b/Å	10.8496(16)
c/Å	13.1050(19)
α/deg.	84.595(6)
β/deg.	72.698(5)
γ/deg.	76.761(5)
V/ Å^3	1361.2(3)
Z	2
T, K	100(2)
λ(MoKα), Å	0.71073
ρcalc, g·cm-3	1.433
μ(MoK$_\alpha$), mm^{-1}	0.761
Flack param.	0.03(1)
R	0.0416
ωR^2	0.0891

Table S3. Main bond distances (Å) and bond angles (deg.) for complex **NiL3**.

	**Molecule A**	**Molecule B**
Ni-N1	1.846(3)	1.855(3)
Ni-N2	1.859(3)	1.853(3)
Ni-O2	1.862(3)	1.853(3)
Ni-O3	1.848(3)	1.857(3)
N1-Ni-N2	86.0(1)	86.0(1)
O2-Ni-O3	86.0(1)	85.7(1)
N1-Ni-O2	93.8(1)	94.1(1)
N2-Ni-O3	94.6(1)	94.6(1)
N1-Ni-O3	174.5(1)	174.6(1)
N2-Ni-O2	176.5(1)	176.5(1)
O3w···O1	2.949(4)	2.845(4)
O3w···O2	2.960(4)	2.963(3)
O3w···O3	2.983(3)	3.069(4)
O3w···O4	2.877(4)	2.939(4)

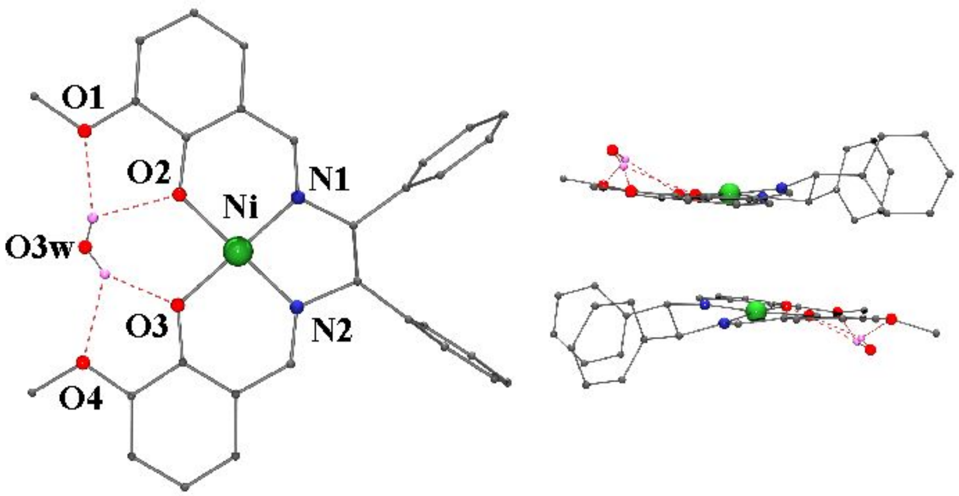

Figure S2. Partially labelled plot for [NiL3(H_2O)]. The unit cell contains two similar but non-equivalent molecules labelled "A" and "B". As has been found in several Salen-type Schiff bases, (CCDC numbers ARARIV; BOCVEX; KOHHAQ; KOHHIY; MABNEK; TUZFAY; VOHSIV; WAVZAU), the Ni^{II} cation is linked in the inner cavity of the ligand whereas a water molecule stablishes two bifurcated H-bonds with the four O-atoms of the ligand.

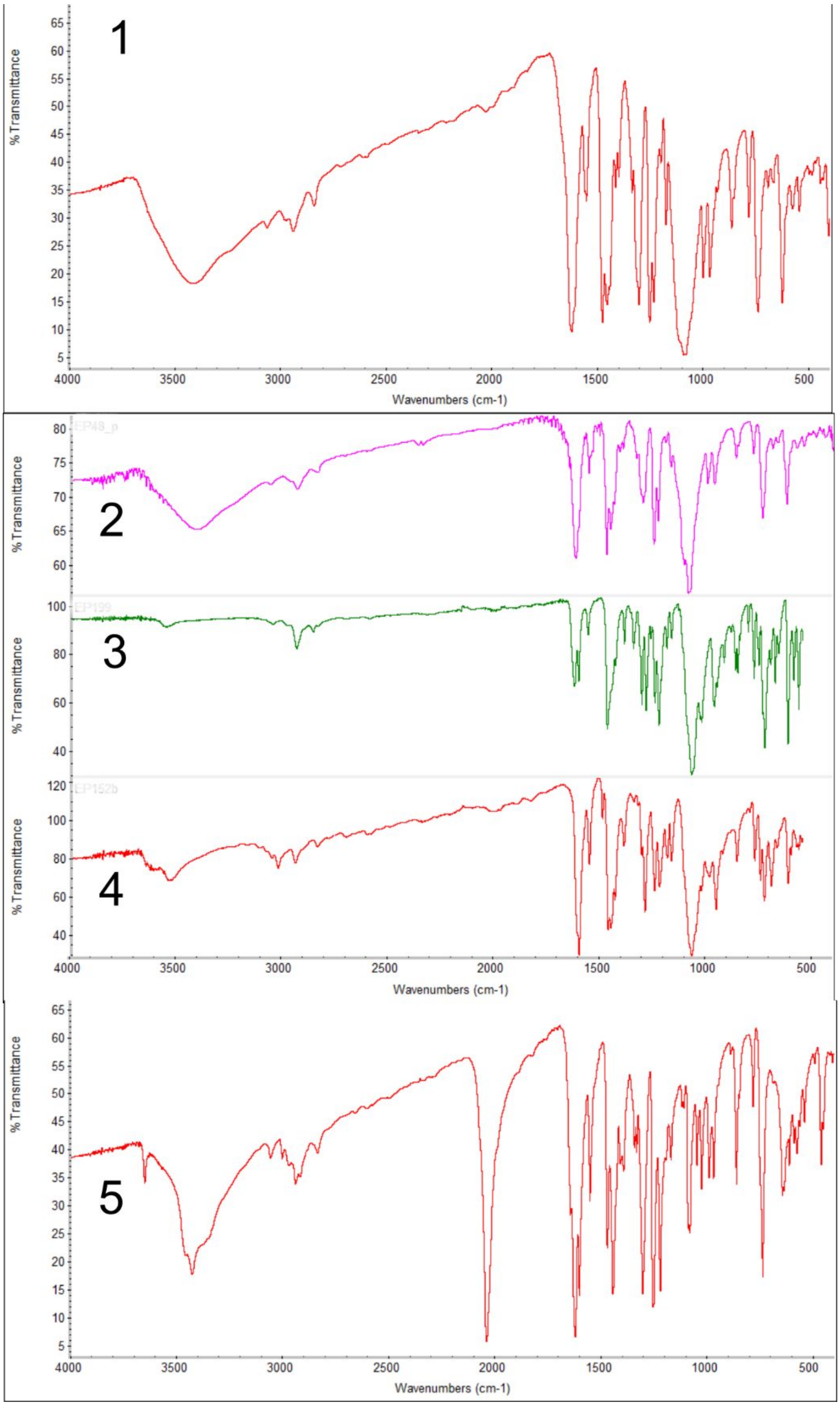

Figure S3. IR spectra for complexes **1** – **5**.

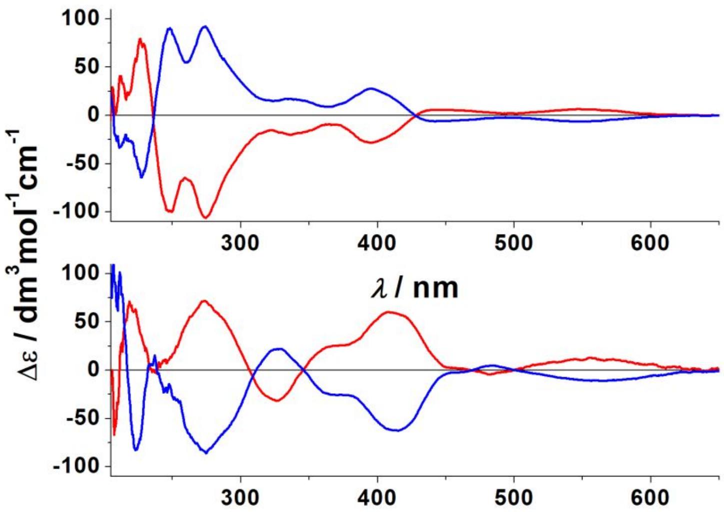

Figure S4. Solution ECD spectra for the pairs of complexes **3*RR***/**3*SS*** (top) and **4*RR***/**4*SS*** (bottom). *RR* enantiomers, red lines, *SS* enantiomers, blue lines. The spectra for **4*RR*** exhibits positive Cotton effect at λ_{max} = 560, 409, 274 and 219 nm and negative bands at 483 and 325 and the same bands with opposite sign for **4*SS***. In the case of **3** positive Cotton effect at λ_{max} = 548, 444, 227 and 211 nm and negative bands at 395, 336, 274 and 247 nm were observed for **3*RR*** and the mirror image for **3*SS***. The absorptions below 300 nm are mainly related with the $\pi \rightarrow \pi^*$ transitions of the aromatic rings and are similar to previously related systems containing the same ligands[33,41] whereas the absorptions at larger wavelengths are related with transitions involving the metallic centres.

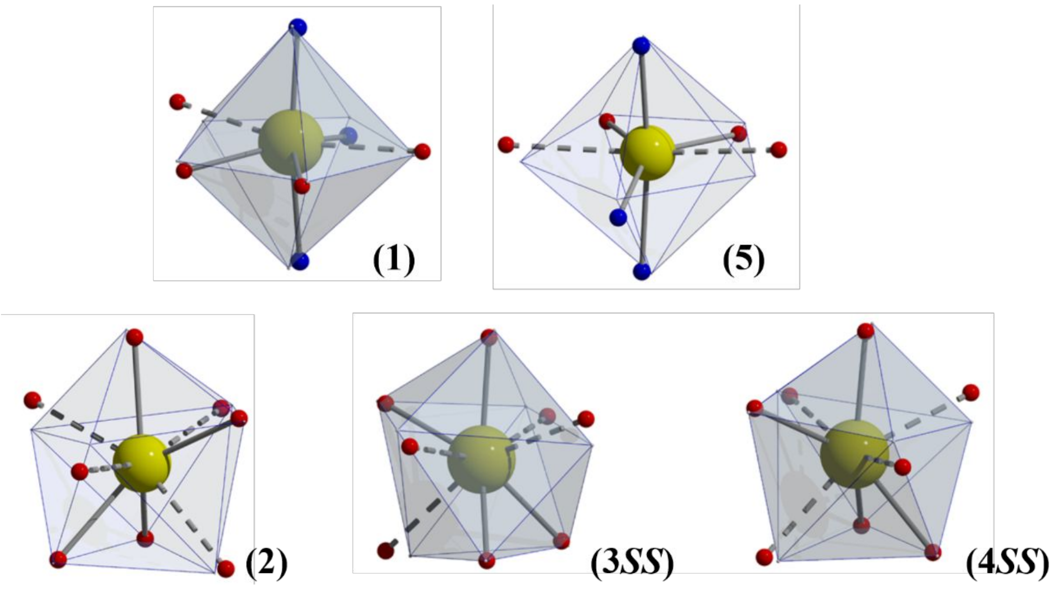

Figure S5. Closest coordination polyhedra around the Mn^{II} cations in complexes **1** – **5**. Due to the low bite of the Schiff base and the large difference in the Mn-O bond lengths they are severely distorted in all cases.

Table S4. Selected bond distances (Å) and angles (deg.) for complex **1**.

Ni2-N3	1.856(1)	Ni2-O9	1.8630(8)
Ni2-N4	1.850(1)	Ni2-O10	1.8498(9)
Ni3-N5	1.844(1)	Ni3-O13	1.8542(9)
Ni3-N6	1.853(1)	Ni3-O14	1.8464(9)
Ni1-N1	1.840(1)	Ni1-O2	1.8449(9)
Ni1-N2	1.840(1)	Ni1-O3	1.8525(8)
Mn-O1	2.5144(9)	Mn-O2	2.2016(8)
Mn-O3	2.1963(9)	Mn-O4	2.5549(9)
Mn-O5	2.1411(9)	Mn-O6	2.1358(9)
Mn-O7	2.1760(8)		
O1-Mn-O2	65.66(3)	O2-Mn-O3	66.96(3)
O3-Mn-O4	65.70(3)	O4-Mn-O7	80.88(3)
O1-Mn-O7	81.55(3)	O5-Mn-O6	174.22(3)
Ni1-O2-Mn	105.54(4)	Ni1-O3-Mn	105.48(4)

Table S5. Parameters for the H-bonds between the neutral [NiL1] fragments and the four O-atoms of the Schiff bases for complex **1**.

	D···A (Å)		D-H···A (deg)
O5···O8	2.930(1)	O5-H5A-O8	131(1)
O5···O9	2.772/1)	O5-H5A-O9	155(1)
O5···O10	2.853(1)	O5-H5B-O10	127(1)
O5···O11	2.845(1)	O5-H5B-O11	160(1)
O6···O12	2.968(1)	O6-H6A-O12	135(1)
O6···O13	2.817(1)	O6-H6A-O13	151(1)
O6···O14	2.849(1)	O6-H6B-O14	126(1)
O6···O15	2.947(1)	O6-H6B-O15	163(1)

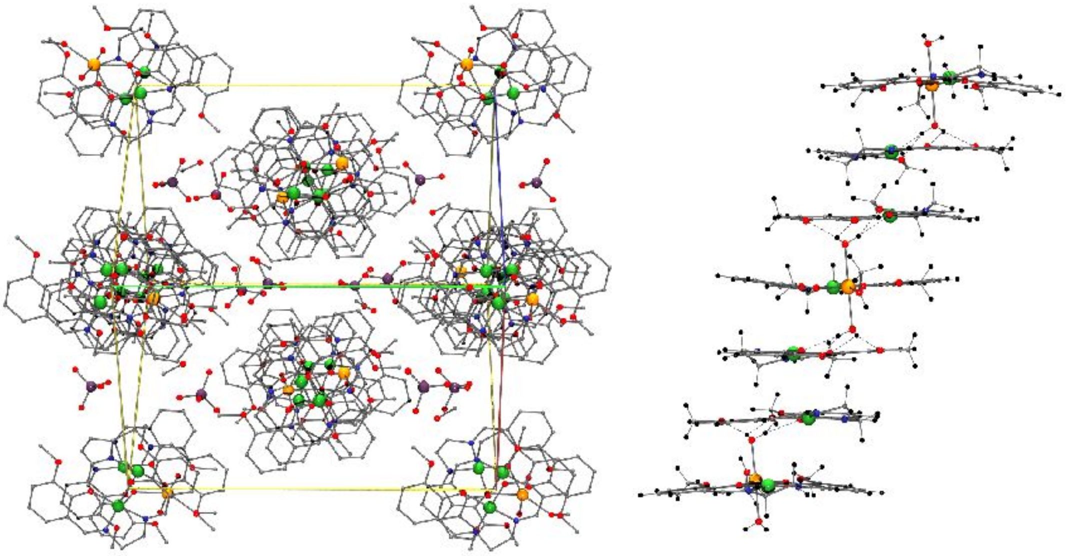

Figure S6. Left, a view of the unit cell along c-axis of complex **1**. Right, pillared arrangement of *sandwich* units of **1**.

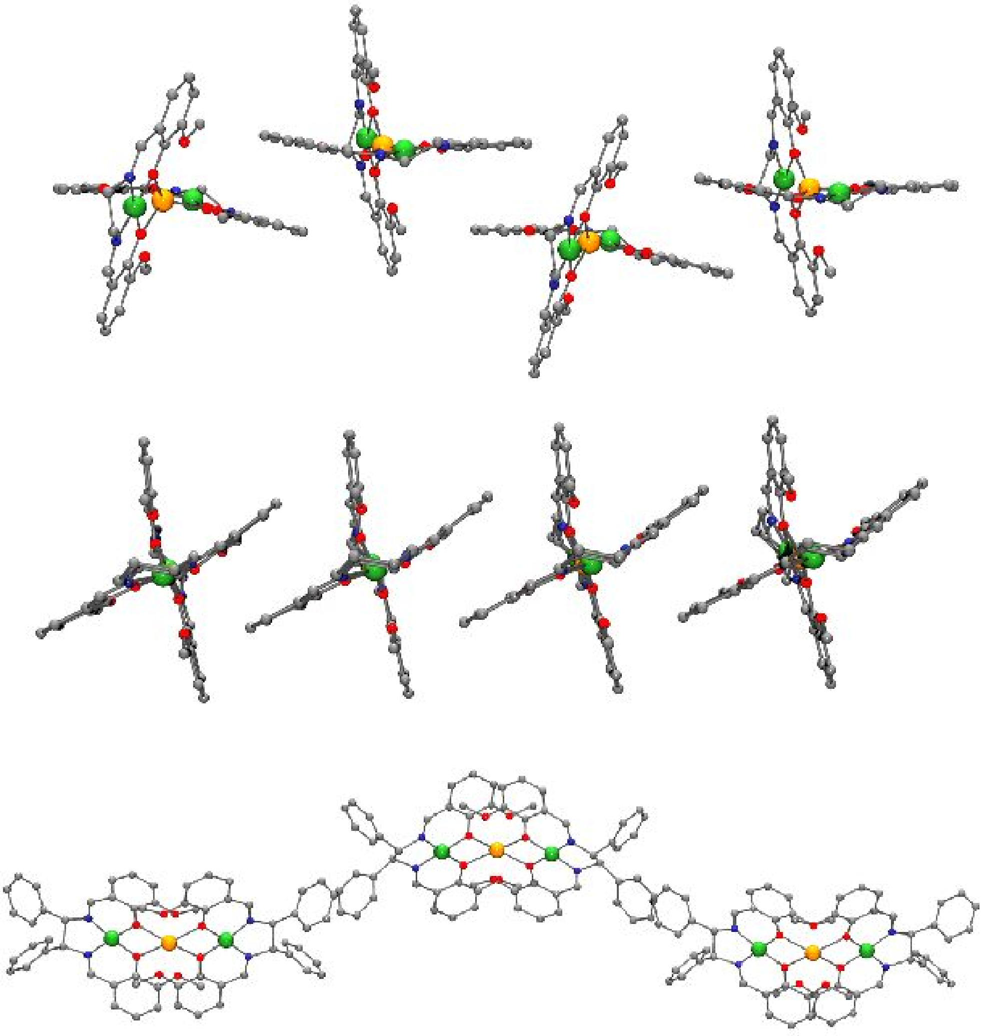

Figure S7. Arrangement of the trimeric units in the network for complexes **2** (top), **3*SS*** (middle) and **4*SS*** (bottom).

Table S6. Selected bond distances (Å) and angles (deg.) for complex **2**.

Ni1-N1	1.838(1)	Ni1-O6	1.8468(8)
Ni1-N2	1.837(1)	Ni1-O7	1.8481(8)
Ni2-N3	1.840(1)	Ni2-O2	1.8521(8)
Ni2-N4	1.845(1)	Ni2-O3	1.8455(8)
Mn-O2	2.1732(8)	Mn-O6	2.1780(8)
Mn-O3	2.1731(8)	Mn-O7	2.1796(8)
Mn-O1	2.520(1)	Mn-O4	2.4790(9)
Mn-O5	2.5527(9)	Mn-O8	2.4238(8)
O2-Mn-O3	67.43(3)	O6-Mn-O7	67.68(3)
O2-Mn-O6	129.00(3)	O2-Mn-O7	136.88(3)
O3-Mn-O6	134.74(3)	O3-Mn-O7	134.15(3)
Ni1-O6-Mn	105.02(4)	Ni1-O7-Mn	104.91(4)
Ni2-O2-Mn	105.44(4)	Ni2-O3-Mn	105.68(4)

Table S7. Selected bond distances (Å) and angles (deg.) for complex **3*SS***.

Ni1-N1	1.844(5)	Ni1-O2	1.854(4)
Ni1-N2	1.851(5)	Ni1-O3	1.848(4)
Ni2-N3	1.855(5)	Ni2-O6	1.851(4)
Ni2-N4	1.855(5)	Ni2-O7	1.842(4)
Mn-O2	2.188(4)	Mn-O6	2.181(4)
Mn-O3	2.159(4)	Mn-O7	2.180(4)
Mn-O1	2.452(4)	Mn-O4	2.514(5)
Mn-O5	2.503(4)	Mn-O8	2.470(4)
O2-Mn-O3	67.8(2)	O6-Mn-O7	66.9(2)
O2-Mn-O6	135.6(2)	O2-Mn-O7	132.2(2)
O3-Mn-O6	130.6(2)	O3-Mn-O7	137.2(2)
Ni1-O2-Mn	104.3(2)	Ni1-O3-Mn	105.6(2)
Ni2-O6-Mn	105.8(2)	Ni2-O7-Mn	106.1(2)

Table S8. Selected bond distances (Å) and angles (deg.) for complexes **4*RR*** and **4*SS***.

	**4*RR***		**4*SS***	
	"A" molecule	"B" molecule	"A" molecule	"B" molecule
Ni-N1	1.833(5)	1.845(5)	1.832(5)	1.834(5)
Ni-N2	1.836(5)	1.832(5)	1.826(5)	1.843(5)
Ni-O2	1.854(4)	1.841(4)	1.833(4)	1.856(5)
Ni-O3	1.841(4)	1.857(4)	1.847(5)	1.838(4)
Mn-O1	2.580(4)	2.558(4)	2.437(5)	2.565(5)
Mn-O2	2.165(4)	2.146(5)	2.149(5)	2.158(4)
Mn-O3	2.153(4)	2.152(4)	2.166(4)	2.146(5)
Mn-O4	2.436(4)	2.462(5)	2.575(5)	2.459(5)
Ni-O2-Mn	105.2(2)	105.0(2)	106.5(2)	104.2(2)
Ni-O3-Mn	106.1(2)	104.2(2)	105.3(2)	105.3(2)
O2-Mn-O3	67.6(1)	68.7(2)	67.2(2)	68.5(2)
O2-Mn-O3'	136.9(2)	130.7(2)	137.0(2)	131.1(2)
O2-Mn-O2'	125.4(2)	139.7(2)	135.7(2)	130.1(3)
O3-Mn-O3'	135.3(2)	130.3(2)	125.9(2)	139.6(3)

Table S9. Main bond (Å) and angles (deg.) for complex **5**.

Ni1-O1	1.842(1)	Ni1-N1	1.844(2)
Ni1-O2	1.848(2)	Ni1-N2	1.840(1)
Mn1-O1	2.250(2)	Mn1-O4	2.538(2)
Mn1-O2	2.221(1)	Mn1-N3	2.217(2)
Mn1-O3	2.554(1)	Mn1-N3'	2.249(2)
Mn-O1-Ni	104.83(6)	Mn1-N6	2.123(2)
Mn-O2-Ni	105.75(6)	Mn-N3-Mn'	103.78(3)

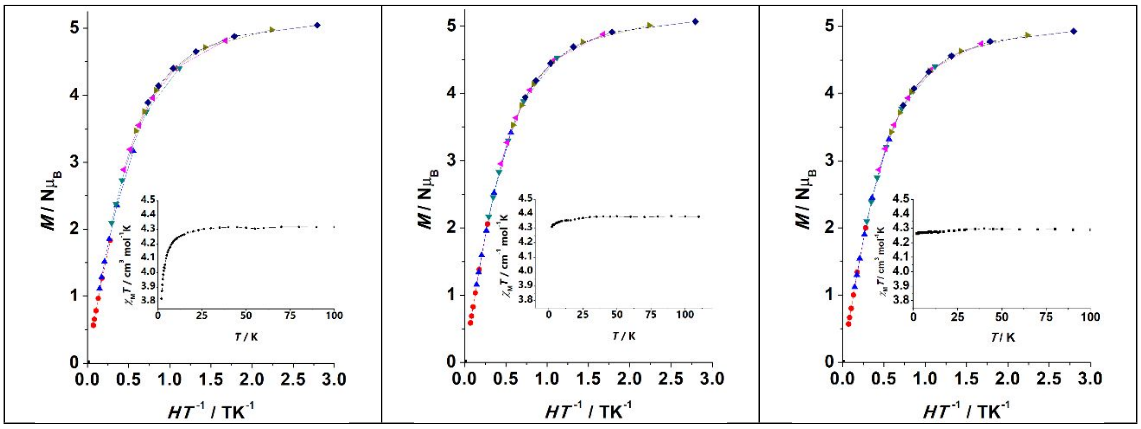

Figure S8. Reduced magnetization and $\chi_M T$ plot (inset) for **2**, (left), **3*SS*** (middle) and **4*SS*** (right).

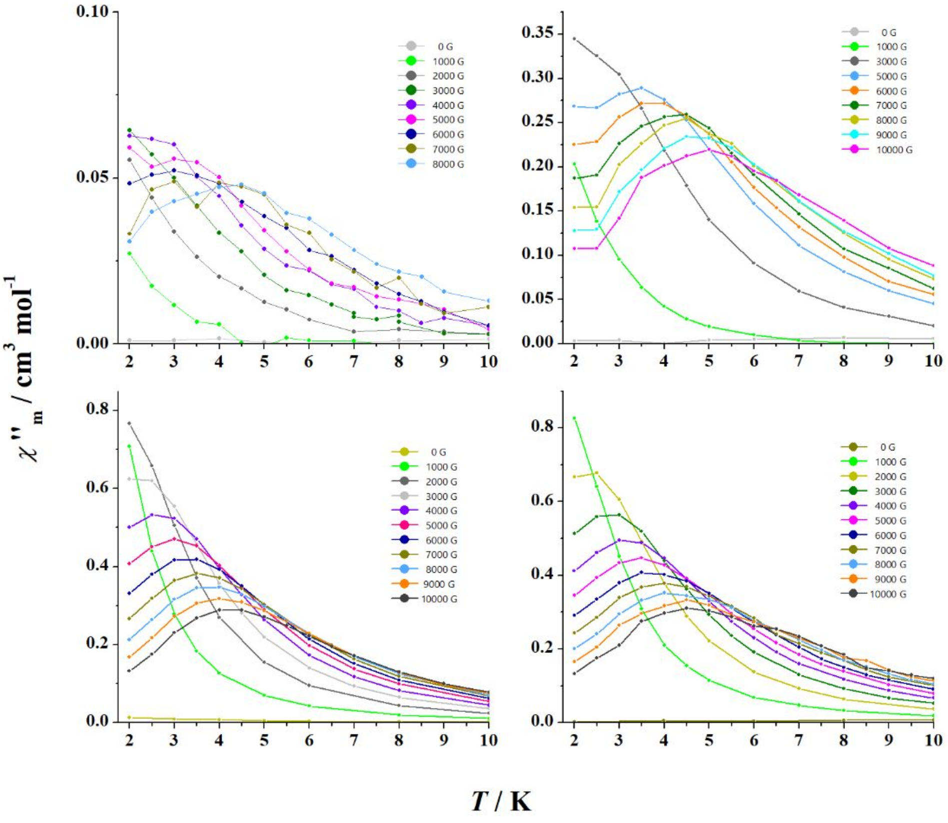

Figure S9. Field dependence of the out-of-phase response of complexes **1** - **4** measured under a fixed frequency of 1488 Hz.

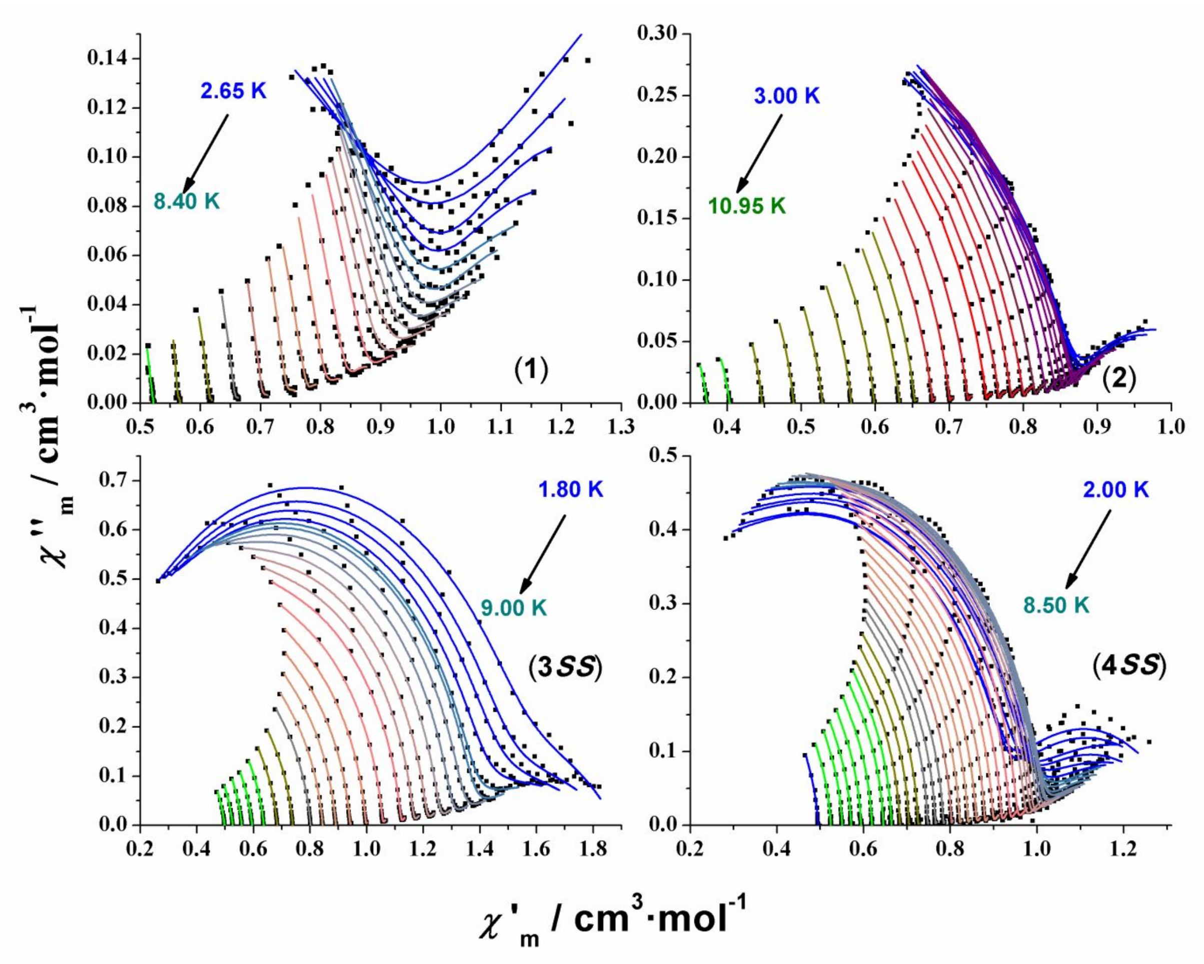

Figure S10. Argand plots for complexes **1** -**4**. Solid lines show the best fit in the temperature range 2.65 – 8.40 for **1**, 3 - 11 K for **2**, 1.8 – 9.0 K for **3*SS*** and 2 – 8.5 K for **4*SS*** to avoid the overlap with the LFT signals.

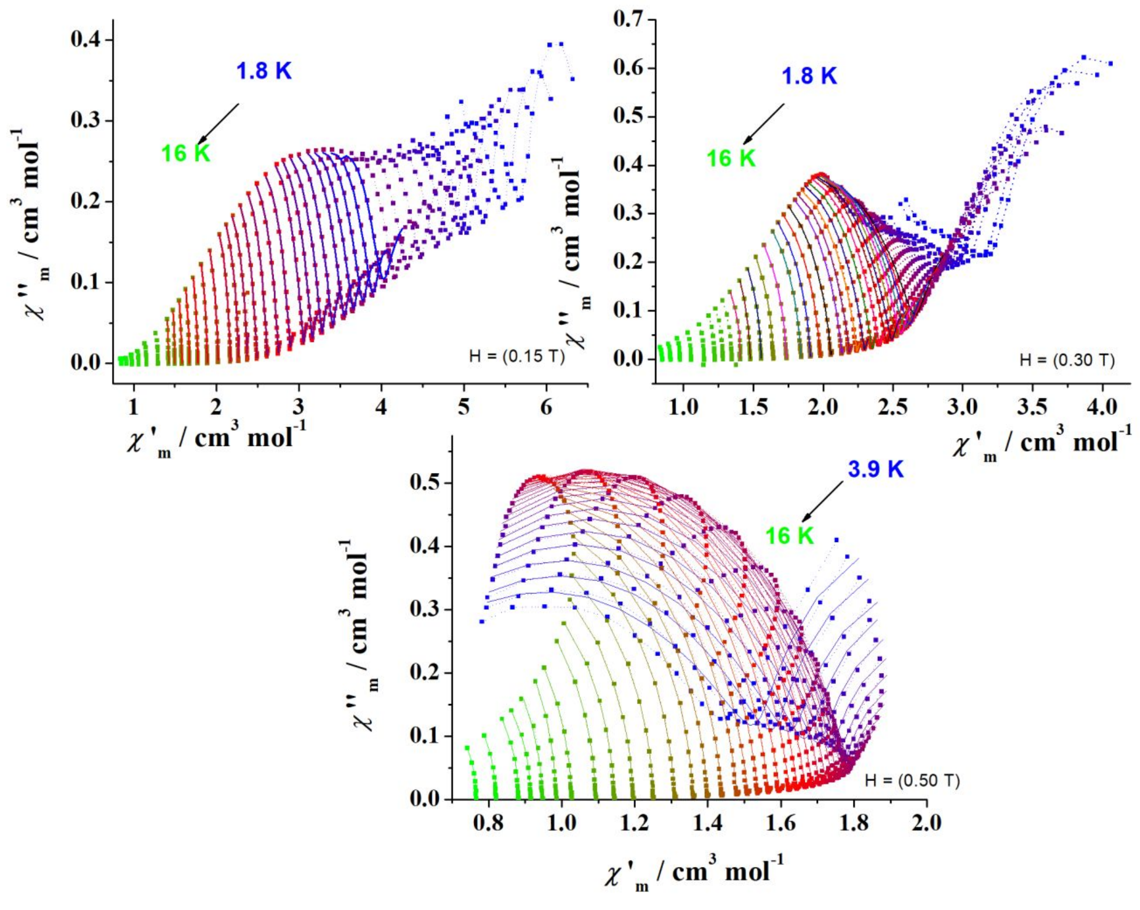

Figure S11. Argand plots for complex **5** under different external fields. Solid lines show the best fit in the temperature range 4 – 7.5 K for 0.15 T, 4 – 9.5 K for 0.30 T and 4.95 – 13 K for 0.50 T to avoid the overlap with the LFT signals.

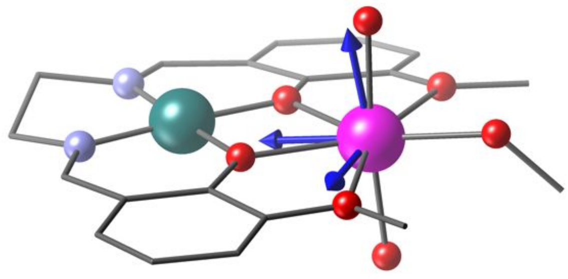

(**1***)

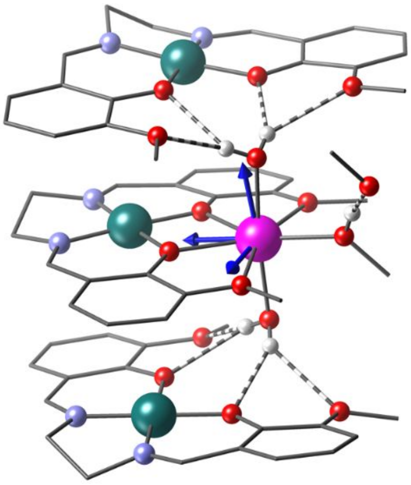

(**1**)

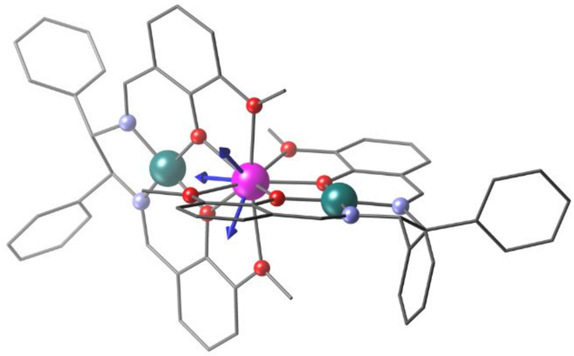

(**4**)

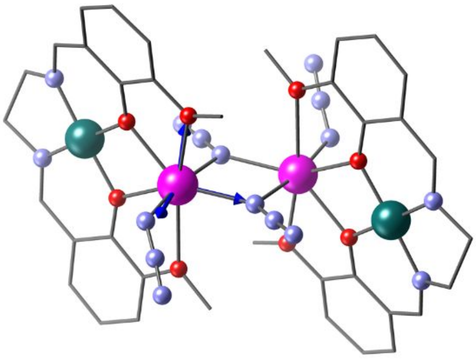

(**5**)

Figure S12. The three components for calculated g-matrix: (a) compound **1** simplified for only Mn(μ-O)2Ni fragment (**1***); (b) compound **1** extended with the two nickel extra units; (c) compound **4** having two Mn(μ-O)$_2$Ni rings, and (d) compound **5** having Ni(μ-O)$_2$Mn$_2$(μ-O)$_2$Mn(μ-O)$_2$Ni core.

File S1. ORCA input files for the CASSCF/NEVPT2 calculations on the reoriented experimental geometry.

(a) Compound **1** simplified for only Mn(μ-O)$_2$Ni fragment.

```
! PATOM def2-TZVPP def2/J def2-TZVPP/C TightSCF Normalprint SlowConv UNO
RIJCOSX

%pal nprocs 7 end

%MaxCore 20000

%scf
MaxDisk 40000
end

%casscf
  nel 5
  norb 5
  mult 6,4,2
  nroots 1,24,75
  shiftup 0.5
  shiftdn 0.5
  trafostep RI
  SwitchConv 0.003
  actorbs dorbs
  printWF true
  nevpt2 true
  rel
    PrintLevel 3
    dosoc true
    nreducedstates 5
    gtensor true
    NDoubGTensor  3
    domagnetization true      # Calculate magnetization (def: false)
    MAGTemperatureMIN 2.0    # minimum temperature (K) for magnetization
    MAGTemperatureMAX 2.0    # maximum temperature (K) for magnetization
    MAGFieldMIN 0.0          # minimum field (Gauss) for magnetization
    MAGFieldMAX 50000.0      # maximum field (Gauss) for magnetization
    MAGNpoints 25           # number of field points for magnetization
    dosusceptibility true     # Calculate susceptiblity (def: false)
    SUSTempMIN 1.0          # minimum temperature (K) for susceptibility
    SUSTempMAX 300.0        # maximum temperature (K) for susceptibility
    SUSNPoints  300                # number of temperature points for
susceptibility
  end
  maxiter 250
  aniso
    doaniso true
  end
end

*xyz +2 6
Mn          0.00000        0.00000        0.00000
C           1.91776       -3.00346       -0.29957
C           3.16289       -0.97757       -0.45431
C           4.32632       -1.64043       -0.76599
C           4.31939        1.14993       -0.57799
C           3.12634        0.43374       -0.37961
```

```
C          4.37069        2.57912       -0.44327
C          3.56700        4.77696        0.02148
C          2.30393        5.49843       -0.39231
C         -0.01856        5.09556       -0.06376
C         -1.23994        4.33531        0.02153
C         -2.46180        5.03884        0.05305
C         -3.66169        4.37333        0.05855
C         -3.68262        2.96933        0.04879
C         -2.49494        2.26123        0.03415
C         -1.24082        2.93133        0.01471
C         -3.63294        0.17420        0.17151
C         -1.53981       -2.84346       -0.56621
H          4.33019       -2.58807       -0.83264
H         -0.09189        6.04283       -0.05859
H         -2.45388        5.98859        0.07088
H         -4.47665        4.86157        0.06907
H         -4.51208        2.50623        0.05227
H          2.19876        6.33017        0.13420
H          2.34747        5.73845       -1.35173
H         -4.22208        0.40635       -0.57645
H         -4.06631        0.41730        1.01623
H         -3.45566       -0.78954        0.16080
H         -2.48747       -2.67591       -0.75155
H         -1.43557       -3.73872       -0.18149
H         -1.02853       -2.78461       -1.40010
H          2.03390       -3.26751       -1.23623
H          1.05534       -3.33360        0.02830
H          2.64070       -3.38677        0.23976
H          0.65801       -0.39221        2.40524
H          0.01884        0.73327        2.46992
H         -0.82028       -0.06517       -2.45997
H          0.42265        0.27886       -2.52036
H         -1.38405       -2.01554        1.11801
H          4.33585        5.10036       -0.51176
H          3.75712        4.94191        0.97886
N          3.35304        3.33677       -0.21469
N          1.16326        4.59393       -0.14632
Ni         1.59446        2.80586       -0.11506
O          1.95223       -1.57195       -0.19807
O          1.95852        0.99719       -0.12945
O         -0.14969        2.19119       -0.00514
O         -2.39426        0.89042        0.04540
O         -0.00000       -0.00000        2.14093
O         -0.10193       -0.18931       -2.12485
O         -1.05598       -1.86837        0.35888
C          5.51116       -0.91457       -0.98586
C          5.51209        0.45398       -0.88257
H          6.31322       -1.37327       -1.20676
H          6.31910        0.93692       -1.01651
H          5.21909        2.99774       -0.52910
*
```

(b) Compound **1** extended with the two nickel extra units.

```
! PATOM def2-TZVPP def2/J def2-TZVPP/C TightSCF Normalprint SlowConv UNO
RIJCOSX

%pal nprocs 7 end
```

```
%MaxCore 20000

%scf
MaxDisk 40000
end

%casscf
  nel 5
  norb 5
  mult 6,4,2
  nroots 1,24,75
  shiftup 0.5
  shiftdn 0.5
  trafostep RI
  SwitchConv 0.003
  actorbs dorbs
  printWF true
  nevpt2 true
  rel
    PrintLevel 3
    dosoc true
    nreducedstates 5
    gtensor true
    NDoubGTensor  3
    domagnetization true      # Calculate magnetization (def: false)
    MAGTemperatureMIN 2.0     # minimum temperature (K) for magnetization
    MAGTemperatureMAX 2.0     # maximum temperature (K) for magnetization
    MAGFieldMIN 0.0           # minimum field (Gauss) for magnetization
    MAGFieldMAX 50000.0       # maximum field (Gauss) for magnetization
    MAGNpoints 25             # number of field points for magnetization
    dosusceptibility true     # Calculate susceptiblity (def: false)
    SUSTempMIN 1.0            # minimum temperature (K) for susceptibility
    SUSTempMAX 300.0          # maximum temperature (K) for susceptibility
    SUSNPoints  300                # number of temperature points for
susceptibility
  end
  maxiter 250
  aniso
    doaniso true
  end
end

*xyz 2 6
Mn         0.00000        0.00000        0.00000
C         -0.10994        5.72286        3.90277
H          1.66189        6.59269        3.48886
H         -0.57055        6.52991        4.10000
H         -1.92043        2.25094        5.24648
C         -3.02280       -1.65207        0.96005
C         -3.08940        0.72847        1.04445
C         -4.34312        0.73807        1.60854
C         -3.03742        3.15243        1.07228
C         -2.39676        1.93401        0.78824
C         -2.42305        4.41474        0.76819
C         -0.77202        5.91101       -0.08484
C          0.73501        5.92899        0.04008
C          2.46793        4.42051       -0.58099
C          3.13357        3.15386       -0.75286
C          4.51083        3.16073       -1.05613
C          5.21881        1.98984       -1.15843
```

C	4.56639	0.75995	-0.97547
C	3.21295	0.73164	-0.69333
C	2.46341	1.93378	-0.56977
C	3.16188	-1.64851	-0.73688
C	0.06658	-3.21071	0.68100
C	1.17572	-2.65377	-3.77648
C	-2.43245	-3.26112	-2.94229
C	-3.89420	-1.49479	-2.33564
C	-4.91686	-2.37775	-2.06457
C	-4.07329	-0.08995	-2.19295
C	-0.84905	4.77291	-3.38012
C	0.12698	5.76064	-3.63923
C	1.42339	5.41679	-3.90409
C	1.79053	4.06072	-3.94140
C	0.85480	3.07846	-3.68218
C	-0.49729	3.40480	-3.37224
C	2.42333	1.31445	-4.04970
C	1.52323	-3.51392	3.80626
C	3.31355	-2.28463	2.81240
C	4.01772	-3.42067	2.46604
C	5.31719	-3.30754	1.95026
C	5.91761	-2.08183	1.84564
C	5.21264	-0.90949	2.20525
C	3.86880	-0.98930	2.62779
C	5.92138	0.33420	2.23719
C	6.24484	2.64969	2.72238
C	5.45519	3.84314	2.24730
C	3.29828	4.63755	2.88884
C	1.90847	4.56629	3.23622
C	1.21047	5.75825	3.53876
C	-0.78163	4.49233	3.98444
C	-0.13028	3.32487	3.67519
C	1.24561	3.32321	3.28782
C	-1.96366	1.93336	4.32044
H	-4.78731	-0.08102	1.79363
H	2.98328	5.20718	-0.71620
H	4.95534	3.98921	-1.19185
H	6.14855	2.01131	-1.35280
H	5.05440	-0.05209	-1.04550
H	-4.76956	-3.31155	-2.15891
H	-0.12132	6.67759	-3.62950
H	2.07320	6.09159	-4.06213
H	2.68613	3.81855	-4.14573
H	3.62277	-4.27753	2.57707
H	5.78370	-4.08667	1.67156
H	6.81141	-2.01833	1.53040
H	6.84995	0.32083	2.03705
H	3.69043	5.50033	2.82249
H	-1.69195	4.46804	4.25508
H	1.12178	6.58098	-0.59662
H	0.99767	6.19321	0.95723
H	3.92186	-1.71104	-0.12130
H	3.48653	-1.68135	-1.66098
H	2.55067	-2.39797	-0.57851
H	0.99419	-3.52643	0.69904
H	-0.52554	-3.95081	0.43201
H	-0.18233	-2.87742	1.56825
H	-3.06804	-1.79078	1.92920
H	-2.50546	-2.37641	0.55026
H	-3.92952	-1.64738	0.58820
H	0.52529	-1.93782	-3.93304

H	1.16363	-3.27722	-4.53261
H	2.07172	-2.26729	-3.68619
H	1.45605	-3.99492	-2.41833
H	-2.57758	-3.73052	-2.09434
H	-1.51422	-3.41549	-3.24805
H	-3.06140	-3.59796	-3.61399
H	3.07586	1.72216	-3.44275
H	2.61236	1.59542	-4.96922
H	2.48451	0.33832	-3.98717
H	2.15443	-3.92019	4.43638
H	1.38814	-4.11632	3.04522
H	0.66660	-3.35903	4.25638
H	7.08126	2.55832	2.20075
H	6.47775	2.74766	3.67958
H	5.80685	4.67355	2.65561
H	5.51495	3.92579	1.26258
H	-2.23069	0.99046	4.31056
H	-2.62072	2.46410	3.82341
H	-1.21523	-0.10159	-2.21018
H	-0.14140	0.55842	-2.51132
H	1.14578	-0.37398	2.29693
H	0.25760	0.54078	2.49997
H	0.00477	-2.47446	-1.05118
H	-1.17285	6.59063	0.51308
H	-1.04052	6.11409	-1.01577
N	-1.23278	4.56244	0.29561
N	1.23146	4.57112	-0.25908
N	5.38141	1.47444	2.52068
N	4.05510	3.62098	2.65992
Ni	0.00000	3.22930	0.00000
Ni	-3.10179	2.54993	-2.61778
Ni	3.58891	1.83092	2.76767
O	-2.38649	-0.39486	0.68570
O	-1.17502	1.83806	0.29632
O	1.17833	1.83014	-0.29301
O	2.46822	-0.41012	-0.51684
O	-0.41290	-0.07628	-2.09935
O	0.40645	-0.13910	2.09205
O	-0.05935	-2.15756	-0.27592
O	-2.64188	-1.86211	-2.75358
O	-3.05230	0.69548	-2.44658
O	-1.32689	2.41888	-3.13747
O	1.10560	1.73385	-3.68426
O	2.04741	-2.27314	3.34275
O	3.10164	0.04608	2.89008
O	1.79217	2.16654	3.02898
O	-0.68151	2.07107	3.71001
O	0.83618	-3.35115	-2.58044
C	-4.96797	1.96160	1.91119
C	-4.33342	3.14747	1.63797
C	-6.17465	-1.91066	-1.64995
C	-6.38099	-0.57145	-1.52426
C	-5.35193	0.35160	-1.79266
C	-5.65016	1.74749	-1.69765
C	-5.28925	4.09708	-1.83673
C	-4.56594	4.89406	-2.89072
C	-2.21473	5.17413	-3.19583
H	-5.83195	1.96693	2.30620
H	-4.76755	3.97032	1.83004
H	-2.93271	5.20042	0.92741
H	-6.87485	-2.52380	-1.45927

```
H         -7.23366       -0.25398       -1.25074
H         -6.51370        1.98836       -1.38332
H         -2.41865        6.09675       -3.29433
H         -5.05944        4.42870       -0.93274
H         -6.26887        4.16691       -1.96067
H         -4.99409        4.77176       -3.77486
H         -4.57278        5.85781       -2.66450
N         -4.84618        2.70230       -2.00288
N         -3.18767        4.37498       -2.90598
*
```

(c) Compound **4** having two Mn(μ-O)$_2$Ni rings, unit 1.

```
! PModel def2-TZVPP def2/J def2-TZVPP/C TightSCF Normalprint SlowConv
UNO RIJCOSX

%pal nprocs 14 end

%MaxCore 20000

%scf
  maxiter 1000
  MaxDisk 40000
end

%casscf
  nel 5
  norb 5
  mult 6,4,2
  nroots 1,24,75
  shiftup 0.5
  shiftdn 0.5
  trafostep RI
  SwitchConv 0.003
  actorbs dorbs
  printWF true
  nevpt2 true
  rel
    PrintLevel 3
    dosoc true
    nreducedstates 5
    gtensor true
    NDoubGTensor  3
    domagnetization true      # Calculate magnetization (def: false)
    MAGTemperatureMIN 2.0    # minimum temperature (K) for magnetization
    MAGTemperatureMAX 2.0    # maximum temperature (K) for magnetization
    MAGFieldMIN 0.0          # minimum field (Gauss) for magnetization
    MAGFieldMAX 50000.0      # maximum field (Gauss) for magnetization
    MAGNpoints 25            # number of field points for magnetization
    dosusceptibility true     # Calculate susceptiblity (def: false)
    SUSTempMIN 1.0          # minimum temperature (K) for susceptibility
    SUSTempMAX 300.0        # maximum temperature (K) for susceptibility
    SUSNPoints  300               # number of temperature points for
susceptibility
  end
  maxiter 250
  aniso
    doaniso true
```

```
  end
end

*xyz 2 6
Mn         0.00000        0.00000        0.00000
C          1.17966       -3.25230        8.51856
C          2.27611       -2.59365        9.01550
C          2.61931       -1.33785        8.53071
C          1.86402       -0.75326        7.52014
H          0.95680       -4.11597        8.84696
H          2.80289       -2.99745        9.69540
H          3.37014       -0.87968        8.88908
H          2.11711        0.09073        7.16619
C          3.59614       -0.69978       -7.83455
C          3.47313        0.11891       -8.89237
C          2.35052        0.86510       -9.02613
H          4.39191       -1.21002       -7.73662
H          4.16528        0.17243       -9.54042
H          2.25526        1.42892       -9.78428
C         -4.29839       -3.40987       -1.51853
C          3.38341       -4.13293        1.96995
C          1.43291       -0.05685       -6.99412
C         -1.30531        1.10871        6.93116
C          2.58686       -0.82025       -6.86857
C         -2.60958        0.63292        6.87979
C          1.31741        0.82483       -8.06311
C         -0.96785        2.05684        7.89064
C         -0.22358       -1.51200       -5.72073
C         -0.06155       -0.83849        5.86140
C         -1.13964       -2.01446       -6.81784
C          0.72397       -1.41218        7.02291
C         -1.11137       -3.35224       -7.16590
C          0.38275       -2.65424        7.52537
C         -2.04190       -3.85569       -8.09316
C         -2.95682       -3.01788       -8.67966
C         -2.98399       -1.66847       -8.35079
C         -2.09684       -1.16604       -7.40528
C         -1.91664       -2.23639       -4.11729
C          1.38714       -2.12870        4.37853
C         -2.53849       -2.38680       -2.83546
C          1.93690       -2.57253        3.13227
C         -3.67515       -3.22179       -2.72288
C          2.84017       -3.66155        3.13484
C         -3.81539       -2.78796       -0.38222
C          3.04634       -3.55187        0.76164
C         -2.69942       -1.96463       -0.45402
C          2.15980       -2.48380        0.72025
C         -2.04129       -1.76337       -1.67580
C          1.58607       -1.98936        1.90040
C         -2.79956       -1.46952        1.88228
C          2.34170       -2.30496       -1.65639
C          1.72173        1.30324       -4.55182
C         -1.29657        2.20166        4.34206
C          0.35531       -0.11147       -5.92953
C         -0.28705        0.67418        5.89585
H         -5.06363       -3.96947       -1.46312
H          3.99242       -4.86126        1.99162
H          2.68889       -1.41933       -6.13785
H         -2.86181       -0.00622        6.22317
H          0.54662       -2.14774       -5.65721
H         -0.96186       -1.27535        5.86207
```

H	-0.46550	-3.93079	-6.77857
H	-0.38782	-3.10365	7.19957
H	-2.03593	-4.78002	-8.31455
H	-3.57428	-3.36159	-9.31483
H	-3.60908	-1.08999	-8.77119
H	-2.13649	-0.25048	-7.15663
H	-2.28080	-2.73226	-4.83998
H	1.63293	-2.60857	5.15962
H	-4.01067	-3.65905	-3.49574
H	3.07283	-4.07220	3.95844
H	-4.24751	-2.92663	0.45206
H	3.42103	-3.88657	-0.04430
H	-2.71645	-2.39894	2.17976
H	2.03531	-3.21615	-1.84371
H	-3.74684	-1.24017	1.78398
H	3.31764	-2.29799	-1.57223
H	-2.38759	-0.87625	2.54626
H	2.07351	-1.71291	-2.39130
H	-0.39030	0.48995	-6.21425
H	0.58436	1.10981	6.11851
N	0.84169	0.35549	-4.60096
N	-0.66707	1.08407	4.51481
N	-0.88313	-1.46768	-4.37495
N	0.57032	-1.11109	4.52928
Ni	-0.04558	-0.37575	-3.17412
Ni	0.00000	-0.00000	3.19661
O	0.82258	0.60481	-1.89180
O	-0.62773	1.00143	1.79567
O	-0.95165	-0.97900	-1.68140
O	0.71487	-0.97291	1.79822
O	-2.13149	-1.30710	0.61739
O	1.74967	-1.84053	-0.42901
C	2.29517	2.19888	1.46507
C	-1.72584	2.49448	-1.74596
C	2.32620	2.11839	-0.92669
C	-1.74105	2.70541	0.63799
C	3.28023	3.12545	-1.06207
C	-2.42564	3.91949	0.64045
C	3.71805	3.51656	-2.30153
C	-2.73995	4.54655	1.81897
C	3.21449	2.92062	-3.44650
C	-2.37660	3.98664	3.03311
C	2.22442	1.90508	-3.34089
C	-1.65861	2.75930	3.06174
C	1.77180	1.53155	-2.08434
C	-1.32598	2.14365	1.86439
H	0.55786	1.38813	-8.14534
H	-0.09487	2.42860	7.91692
H	3.24849	2.00100	1.57509
H	-2.70033	2.51790	-1.84554
H	1.79502	1.82975	2.22255
H	-1.33889	1.93041	-2.44759
H	2.16536	3.17021	1.43066
H	-1.36787	3.40402	-1.82412
H	3.63122	3.54796	-0.28638
H	-2.67689	4.31927	-0.18446
H	4.37370	4.19991	-2.37629
H	-3.21278	5.37034	1.80353
H	3.52804	3.19009	-4.30166
H	-2.60469	4.42165	3.84611
H	2.06344	1.62843	-5.37587

```
H         -1.53917        2.69343        5.11722
O          1.81778        1.60589        0.23737
O         -1.38603        1.95336       -0.45039
C         -3.54722        1.10306        7.81041
C         -3.21763        1.98737        8.76632
C         -1.94777        2.45500        8.82743
H         -4.44295        0.78924        7.76180
H         -3.86778        2.28005        9.39365
H         -1.71013        3.06498        9.51533
*
```

(d) Compound **4** having two Mn(μ-O)$_2$Ni rings, unit 2.

```
! PModel def2-TZVPP def2/J def2-TZVPP/C TightSCF Normalprint SlowConv
UNO RIJCOSX

%pal nprocs 14 end

%MaxCore 20000

%scf
  maxiter 1000
  MaxDisk 40000
end

%casscf
  nel 5
  norb 5
  mult 6,4,2
  nroots 1,24,75
  shiftup 0.5
  shiftdn 0.5
  trafostep RI
  SwitchConv 0.003
  actorbs dorbs
  printWF true
  nevpt2 true
  rel
    PrintLevel 3
    dosoc true
    nreducedstates 5
    gtensor true
    NDoubGTensor  3
    domagnetization true      # Calculate magnetization (def: false)
    MAGTemperatureMIN 2.0    # minimum temperature (K) for magnetization
    MAGTemperatureMAX 2.0    # maximum temperature (K) for magnetization
    MAGFieldMIN 0.0           # minimum field (Gauss) for magnetization
    MAGFieldMAX 50000.0       # maximum field (Gauss) for magnetization
    MAGNpoints 25            # number of field points for magnetization
    dosusceptibility true     # Calculate susceptiblity (def: false)
    SUSTempMIN 1.0         # minimum temperature (K) for susceptibility
    SUSTempMAX 300.0       # maximum temperature (K) for susceptibility
    SUSNPoints 300               # number of temperature points for
susceptibility
  end
  maxiter 350
  aniso
    doaniso true
```

```
  end
end

*xyz 2 6
Mn          0.00000         0.00000         0.00000
C           2.06452         1.00859         7.76284
C           2.50606         1.92232         8.70548
C           1.79038         3.14750         8.80557
H           2.53842         0.19316         7.65313
H           3.25396         1.73979         9.26016
H           2.09590         3.81076         9.41242
C          -1.15442        -2.88672         8.38096
C          -2.33172        -2.54792         9.00964
C          -3.04179        -1.44460         8.62717
C          -2.56231        -0.62292         7.56966
H          -0.68332        -3.66974         8.63964
H          -2.65348        -3.08923         9.72078
H          -3.85620        -1.22663         9.06663
H          -3.06311         0.13293         7.28293
C          -0.85937         2.06450        -6.79098
C           0.95192         1.25353         6.97428
C          -1.98143         1.97005        -7.59829
C          -2.36861         3.00510        -8.43327
C          -1.58564         4.19217        -8.40010
C          -0.45851         4.29586        -7.62998
C           0.67312         3.39780         8.05500
C          -0.08854         3.23363        -6.82480
C           0.24716         2.45286         7.13894
C           0.89080         0.47871        -5.79296
C          -0.88060        -0.11304         5.81316
C           1.33890        -0.23461        -7.03263
C          -1.37146        -0.93246         6.96815
C           0.56786        -1.29071        -7.55287
C          -0.66190        -2.08061         7.36663
C          -2.40585         2.02068         1.91170
C           2.52029         2.08197        -1.68505
C           1.73143        -1.40682        -4.48016
C          -1.82068        -1.79297         4.30339
C           1.98212        -2.19817        -3.31251
C          -2.11138        -2.43583         3.05651
C           2.73554        -3.38567        -3.41749
C          -2.92995        -3.58395         3.03246
C           3.03415        -4.09502        -2.29947
C          -3.26409        -4.14824         1.84409
C           2.63668        -3.64719        -1.02081
C          -2.83842        -3.58518         0.62127
C           1.91388        -2.49342        -0.91770
C          -2.05230        -2.46903         0.64356
C           1.55742        -1.76320        -2.05263
C          -1.65930        -1.88912         1.85086
C          -2.29163         2.41639        -0.43490
C           2.42103         2.22272         0.69118
C           1.80401        -2.63229         1.48585
C          -1.94295        -2.34841        -1.76100
C          -3.25225         3.40024        -0.35198
C           3.43510         3.15481         0.71410
C          -3.67748         4.05691        -1.51337
C           3.89260         3.65500         1.93940
C          -3.15241         3.70651        -2.73023
C           3.34513         3.20253         3.11191
C          -2.19149         2.67733        -2.82167
```

C	2.32822	2.22454	3.09247
C	-1.75956	2.01551	-1.66986
C	1.86372	1.71855	1.87615
C	-1.73563	2.28702	-4.14308
C	1.84732	1.71710	4.36428
C	-0.54650	0.96487	-5.81047
C	0.58144	0.28760	5.87966
H	-2.50025	1.17511	-7.57755
H	-3.12379	2.92658	-9.00237
H	-1.85197	4.93415	-8.92932
H	0.06391	5.08873	-7.64843
H	0.19552	4.21162	8.16222
H	0.69317	3.29821	-6.29072
H	-0.52811	2.61918	6.61762
H	1.48208	1.27660	-5.67155
H	-1.42625	0.72498	5.78212
H	-0.26478	-1.51377	-7.15158
H	0.15829	-2.30408	6.94067
H	-2.26527	2.96996	2.10886
H	2.43329	3.05352	-1.77610
H	-1.98943	1.47637	2.61306
H	2.07642	1.64215	-2.44078
H	-3.36679	1.83186	1.87986
H	3.46915	1.83768	-1.67714
H	2.15321	-1.68378	-5.28548
H	-2.25967	-2.13302	5.07462
H	3.03525	-3.68608	-4.26679
H	-3.24850	-3.95890	3.84445
H	3.51941	-4.90911	-2.37787
H	-3.79408	-4.93777	1.83389
H	2.86878	-4.14180	-0.24324
H	-3.09526	-3.97753	-0.20530
H	1.50679	-2.09932	2.25285
H	-1.61420	-1.75160	-2.46559
H	2.77361	-2.76947	1.54072
H	-2.91850	-2.42461	-1.82766
H	1.35078	-3.50066	1.49591
H	-1.53865	-3.23423	-1.86797
H	-3.62442	3.63231	0.49057
H	3.82218	3.45716	-0.09893
H	-4.32834	4.74647	-1.45734
H	4.58095	4.30930	1.95761
H	-3.43615	4.16004	-3.51566
H	3.65122	3.55037	3.94161
H	-2.12083	2.73659	-4.88462
H	2.25464	2.06021	5.14961
H	-1.12963	0.18425	-6.03036
H	1.11960	-0.54367	6.01050
N	-0.85549	1.38449	-4.39745
N	0.91760	0.84237	4.52047
N	0.99115	-0.36064	-4.54255
N	-1.02360	-0.80279	4.47828
Ni	0.00974	0.34925	-3.15380
Ni	0.00000	-0.00000	3.17309
O	-1.81002	1.70062	0.64055
O	1.90369	1.65755	-0.45503
O	-0.88615	1.02993	-1.67739
O	0.93687	0.78820	1.77783
O	0.83731	-0.66199	-1.86145
O	-0.87851	-0.81529	1.77984
O	1.48756	-1.93901	0.26980

```
O         -1.59219       -1.81184       -0.47701
C          1.01793       -2.00639       -8.65128
C          2.21416       -1.66641       -9.24245
C          2.98327       -0.65307       -8.74305
C          2.54696        0.07259       -7.60006
H          0.50483       -2.72879       -8.99312
H          2.50752       -2.14320      -10.00984
H          3.80988       -0.43366       -9.15835
H          3.08811        0.76328       -7.23342
*
```

(e) Binuclear compound **5** having $Mn_2(\mu\text{-}O)_2$ core (by replacing a Mn atom by Zn one).

```
! PModel def2-TZVPP def2/J def2-TZVPP/C TightSCF Normalprint SlowConv
UNO RIJCOSX

%pal nprocs 14 end

%MaxCore 20000

%scf
  maxiter 1000
  MaxDisk 40000
end

%casscf
  nel 5
  norb 5
  mult 6,4,2
  nroots 1,24,75
  shiftup 0.5
  shiftdn 0.5
  trafostep RI
  SwitchConv 0.003
  actorbs dorbs
  printWF true
  nevpt2 true
  rel
    PrintLevel 3
    dosoc true
    nreducedstates 5
    gtensor true
    NDoubGTensor  3
    domagnetization true      # Calculate magnetization (def: false)
    MAGTemperatureMIN 2.0    # minimum temperature (K) for magnetization
    MAGTemperatureMAX 2.0    # maximum temperature (K) for magnetization
    MAGFieldMIN 0.0           # minimum field (Gauss) for magnetization
    MAGFieldMAX 50000.0       # maximum field (Gauss) for magnetization
    MAGNpoints 25             # number of field points for magnetization
    dosusceptibility true     # Calculate susceptiblity (def: false)
    SUSTempMIN 1.0          # minimum temperature (K) for susceptibility
    SUSTempMAX 300.0        # maximum temperature (K) for susceptibility
    SUSNPoints 300                 # number of temperature points for
susceptibility
  end
  maxiter 250
  aniso
    doaniso true
```

```
  end
end

*xyz 0 6
Mn         0.00000       0.00000       -0.00000
Zn         0.00000       0.00000        3.51415
C          0.83767       5.22300        5.63371
C         -0.09242       4.53739        4.84361
H          2.61696       5.10659        6.55655
H          0.66113       6.11935        5.89460
H         -0.88697       4.97017        4.55513
H         -2.41803       3.26873        4.01839
C         -0.83767      -5.22300       -2.11957
C          0.09242      -4.53739       -1.32946
H         -2.61696      -5.10659       -3.04240
H         -0.66113      -6.11935       -2.38046
H          0.88697      -4.97017       -1.04098
H          2.41803      -3.26873       -0.50425
C          1.16669       3.28086        0.84062
C          1.87633      -2.44790        4.49422
C          0.92718      -3.25700        3.83254
C          1.05456      -4.63255        3.80679
C          2.15915      -5.23568        4.41740
C          3.10762      -4.47940        5.03275
C          2.99133      -3.07118        5.08941
C          4.01565      -2.30940        5.75358
C          5.15864      -0.36672        6.57032
C          5.25699       1.03134        6.05498
C          3.54797       2.71118        6.01917
C          2.27059       3.28574        5.67301
C          1.99164       4.62106        6.03148
C          0.15562       3.22900        4.48734
C          1.32332       2.56450        4.92347
C         -1.87494       3.01704        3.24243
H          1.55342       3.89128        0.17821
H          1.85720       2.66291        1.15883
H          0.81735       3.79761        1.59632
H          0.39561      -5.16505        3.37755
H          2.24761      -6.18122        4.40134
H          3.85939      -4.90371        5.43088
H          4.96307      -0.36337        7.54078
H          6.01048      -0.84849        6.42162
H          5.78965       1.05395        5.22161
H          5.69513       1.61477        6.72439
H          4.76801      -2.73697        6.15367
H          4.22189       3.29330        6.39285
H         -1.69077       3.81345        2.70021
H         -2.36035       2.35980        2.70251
N          4.05643      -1.02543        5.82328
N          3.88152       1.49593        5.79584
N          1.37720       0.04915        1.73679
N          2.57054      -0.04945        1.83492
N          3.69732      -0.16790        1.95925
N         -1.50185      -0.21019        5.00047
N         -2.47810      -0.85007        5.03465
N         -3.45428      -1.47319        5.13591
Ni         2.78470       0.14974        5.18822
O          1.64360      -1.14374        4.54074
O          1.46248       1.28885        4.57906
O         -0.10548      -2.53999        3.27313
O         -0.64068       2.44938        3.68901
```

C	-1.16669	-3.28086	2.67353
C	-1.87633	2.44790	-0.98007
C	-0.92718	3.25700	-0.31839
C	-1.05456	4.63255	-0.29265
C	-2.15915	5.23568	-0.90325
C	-3.10762	4.47940	-1.51860
C	-2.99133	3.07118	-1.57526
C	-4.01565	2.30940	-2.23943
C	-5.15864	0.36672	-3.05617
C	-5.25699	-1.03134	-2.54084
C	-3.54797	-2.71118	-2.50502
C	-2.27059	-3.28574	-2.15887
C	-1.99164	-4.62106	-2.51734
C	-0.15562	-3.22900	-0.97319
C	-1.32332	-2.56450	-1.40932
C	1.87494	-3.01704	0.27172
H	-1.55342	-3.89128	3.33593
H	-1.85720	-2.66291	2.35532
H	-0.81735	-3.79761	1.91783
H	-0.39561	5.16505	0.13660
H	-2.24761	6.18122	-0.88719
H	-3.85939	4.90371	-1.91674
H	-4.96307	0.36337	-4.02663
H	-6.01048	0.84849	-2.90748
H	-5.78965	-1.05395	-1.70746
H	-5.69513	-1.61477	-3.21024
H	-4.76801	2.73697	-2.63952
H	-4.22189	-3.29330	-2.87870
H	1.69077	-3.81345	0.81394
H	2.36035	-2.35980	0.81164
N	-4.05643	1.02543	-2.30914
N	-3.88152	-1.49593	-2.28170
N	-1.37720	-0.04915	1.77735
N	-2.57054	0.04945	1.67922
N	-3.69732	0.16790	1.55490
N	1.50185	0.21019	-1.48633
N	2.47810	0.85007	-1.52051
N	3.45428	1.47319	-1.62177
Ni	-2.78470	-0.14974	-1.67407
O	-1.64360	1.14374	-1.02659
O	-1.46248	-1.28885	-1.06491
O	0.10548	2.53999	0.24102
O	0.64069	-2.44938	-0.17487

*
